# Isolation and Biological Evaluation of Prenylated Flavonoids from* Maclura pomifera*

**DOI:** 10.1155/2018/1370368

**Published:** 2018-01-14

**Authors:** Yerkebulan Orazbekov, Mohamed A. Ibrahim, Serjan Mombekov, Radhakrishnan Srivedavyasasri, Ubaidilla Datkhayev, Bauyrzhan Makhatov, Narayan D. Chaurasiya, Babu L. Tekwani, Samir A. Ross

**Affiliations:** ^1^National Center for Natural Products Research, University of Mississippi, University, MS 38677, USA; ^2^South-Kazakhstan State Pharmaceutical Academy, Al-Farabi Square, Shymkent 160019, Kazakhstan; ^3^Department of Chemistry of Natural Compounds, National Research Center, Dokki, Cairo 12622, Egypt; ^4^Kazakh National Medical University, Almaty 050000, Kazakhstan; ^5^Department of Biomolecular Science, University of Mississippi, University, MS 38677, USA

## Abstract

Phytochemical analysis of the ethanolic extract of* Maclura pomifera* fruits yielded four new compounds (**I**–**IV**) along with eleven known compounds (**V**–**XV**). The crude extract exhibited significant activity towards cannabinoid receptors (CB1: 103.4% displacement; CB2: 68.8% displacement) and possibly allosteric interaction with *δ* and *μ* opioid receptors (−49.7 and −53.8% displacement, resp.). Compound** I** was found to be possibly allosteric for *κ* and *μ* opioid receptors (−88.4 and −27.2% displacement, resp.) and showed moderate activity (60.5% displacement) towards CB1 receptor. Compound** II** exhibited moderate activity towards cannabinoid receptors CB1 and CB2 (47.9 and 42.3% displacement, resp.). The known compounds (**V**–**VIII**) exhibited prominent activity towards cannabinoid receptors: pomiferin (**V**) (IC_50_ of 2.110 and 1.318 *μ*M for CB1 and CB2, resp.), auriculasin (**VI**) (IC_50_ of 8.923 *μ*M for CB1), warangalone (**VII**) (IC_50_ of 1.670 and 4.438 *μ*M for CB1 and CB2, resp.), and osajin (**VIII**) (IC_50_ of 3.859 and 7.646 *μ*M for CB1 and CB2, resp.). The isolated compounds were also tested for inhibition of human monoamine oxidase-A and monoamine oxidase-B enzymes activities, where all the tested compounds showed fewer inhibitory effects on MAO-A compared to MAO-B activities: auriculasin (**VI**) (IC_50_ of 1.91 and 45.98 *μ*M for MAO-B and MAO-A, resp.).

## 1. Introduction

*Maclura pomifera* L. (*Maclura aurantiaca* Syn., Moraceae family) is a native southwestern American plant commonly known as Osage orange. Osage orange typically grows in sunny areas and can grow in a wide range of soil conditions [1]. Worldwide, various* Maclura* species are used in folk medicine. Native Americans used* M. pomifera* for the treatment of cancer [2]. In Bolivia, the plant sap is used for the treatment of tooth pain, and the bark and leaves are used for uterine hemorrhage [3]. Comanche Indians in North America used the Osage orange roots decoction to treat sore eyes [4].* M. pomifera* and its components possess several biological activities including cytotoxic, antitumor, antibacterial, estrogenic, antifungal, antiviral, and antimalarial activities [5–13]. Recently, isoflavones isolated from Osage orange have been demonstrated to protect brain cells, or neurons, from the toxic effect of amyloid beta peptide, which is believed to be responsible for the degeneration of neurons in Alzheimer's disease patients. However, the mechanisms by which isoflavones block the toxicity of amyloid beta peptide are unknown [14].* M*.* pomifera* produces several secondary metabolites belonging to different chemical classes including prenylated flavonoids. The prenylated flavonoids possess different biological activities such as antifungal, antibacterial, antitumor, and antioxidant activities. The wide range of bioactivities of these compounds is attributed to the prenylation on the flavonoids, which in turn increases their lipophilicity and membrane permeability [15]. In this report, we have examined* M. pomifera *growing in Kazakhstan which has never been exposed to extensive phytochemical or biological studies. We present the isolation and characterization of four new and eleven known metabolites from the fruits of* M. pomifera *growing in Kazakhstan and their accompanying cannabinoid, opioid, and MAO receptors activities.

## 2. Materials and Methods

### 2.1. Apparatus, Materials, and Chemicals

A Bruker model AMX 500 NMR and 400 NMR spectrometers operating on a standard pulse system were used to acquire ^1^H and ^13^C NMR and 2D spectra. The instruments ran at 500 and 400 MHz for ^1^H while they ran at 125 and 100 MHz for ^13^C. CDCl_3_, DMSO-*d*_*6*_, and acetone-*d*_*6*_ were used as NMR solvents, and TMS was used as an internal standard. ESI-MS data were recorded on Thermo Orbitrap Fusion (Thermo Scientific). Samples were analyzed in the negative mode of ionization. Samples were directly infused at 3 uL/min. Mass was analyzed in Orbitrap (mass error on the instrument <2 ppm). ESI-MS data were obtained on a Micromass Q-Tof micromass spectrometer. FTMS-ESI was analyzed on Thermo Orbitrap Fusion (Thermo Scientific). The sample was analyzed in the negative mode of ionization. Mass was analyzed in Orbitrap (mass error on the instrument <2 ppm). TLC was performed on precoated silica gel GF254 plates and Column Chromatography was performed on silica gel (200–300 mesh) and Sorbadex-LH20 (Sorbent Technologies, Atlanta, GA, USA). The recombinant human monoamine oxidase-A and monoamine oxidase-B enzymes were obtained from BD Biosciences (Bedford, MA, USA). Kynuramine, clorgyline, phenelzine, deprenyl, and DMSO were procured from Sigma Chemical Company (St. Louis, MO, USA).

### 2.2. Plant Material

Fresh fruits of* M. pomifera *(L.) (20 Kg) were purchased from Shymkent, Kazakhstan, in October 2015. A voucher specimen of* M. pomifera *was identified by Dr. Kulpan Orynbasarova and deposited at the Department of Pharmacognosy and Chemistry, South-Kazakhstan State Pharmaceutical Academy, Shymkent, Kazakhstan, with an index number “MA-777.”

### 2.3. Extraction and Isolation

Fresh fruits were cut into small pieces and macerated with ethanol (2 × 50 L, 48 h each) at 25°C. The combined extracts were concentrated under reduced pressure to yield crude extract (1 Kg). The extract showed a yellow precipitate which was filtered and weighed (85 g). Eighty grams of the precipitate was loaded on silica gel and fractionated using DCM-MeOH gradient to yield 6 fractions (A1–A6). Fraction A1 (4 g) was loaded on a silica gel column, where the elution was completed using DCM-MeOH gradient to yield stigmasterol (**XIV**, 15 mg) and *β*-sitosterol (**XV**, 20 mg). Fraction A2 (7 g) was loaded on Sorbadex-LH20 and eluted with MeOH-H_2_O to yield pomiferin (**V**, 500 mg) [16], auriculasin (**VI**, 100 mg) [16], warangalone (**VII**, 50 mg) [17], and osajin (**VIII**, 500 mg) [16].

**Figure 1:**
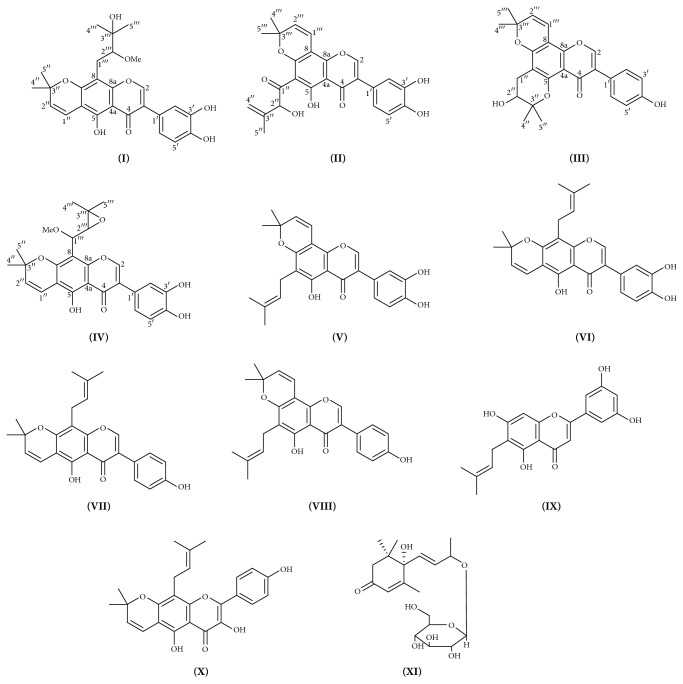
Structures of the selected compounds from* M. pomifera*.

Chromatographic purification of fraction A3 (1.5 g) on Sorbadex-LH20 using MeOH-H_2_O gradient yielded compound** I** (10 mg), compound** II **(5 mg), artocarpesin (**IX**, 7 mg) [16], compound** III** (10 mg), compound** IV** (7 mg), kaempferol-7-*O*-*β*-*D*-glucoside (**XII**, 8 mg) [16], dihydrokaempferol-7-*O*-*β*-D-glucoside (**XIII**, 10 mg) [16], tonkinensisol (**X**, 5 mg) [18], and corchoionoside B (**XI**, 15 mg) [19]. The structures of the isolated compounds (Figure 1) were established using NMR (1D, 2D), IR, and mass spectral data.

#### 2.3.1. 3-(3,4-Dihydroxyphenyl)-5-hydroxy-10-(3-hydroxy-2-methoxy-3-methylbutyl)-8,8-dimethylpyrano[3,2-g]chromen-4(8H)-one (**I**), Named Kazosajin I

IR (neat) cm^−1^: 2927, 1593, 1250, 1120. HR-FTMS:* m*/*z* [M + Na]^+^ calcd. for C_26_H_28_NaO_8_: 491.1682; found: 491.1690. ^1^H NMR (500 MHz, DMSO-*d*6, Supporting Information (available here)): Table 1. ^13^C NMR (125 MHz, DMSO-*d*6, Supporting Information (available here)): Table 2.

#### 2.3.2. 3-(3,4-Dihydroxyphenyl)-5-hydroxy-6-(2-hydroxy-3-methylbut-3-enoyl)-8,8-dimethylpyrano[2,3-f]chromen-4(8H)-one (**II**), Named Kazosajin II

IR (neat) cm^−1^: 2927, 1649, 1438, 1263, 1120 cm^−1^. HR-FTMS:* m*/*z* [M − H]^−^ calcd. for C_25_H_23_O_8_: 451.1393; found: 451.1415. ^1^H NMR (500 MHz, DMSO-*d*6, Supporting Information (available here)): Table 1. ^13^C NMR (125 MHz, DMSO-*d*6, Supporting Information (available here)): Table 2.

#### 2.3.3. 11-Hydroxy-7-(4-hydroxyphenyl)-2,2,10,10-tetramethyl-11,12-dihydro-2H-dipyrano[2,3-f: 2′,3′-h]chromen-8(10H)-one (**III**), Named Kazosajin III

IR (neat) cm^−1^: 2927, 1647, 1578, 1438, 1258, 1194, 1118. HR-FTMS:* m*/*z* [M + H]^+^ calcd. for C_25_H_25_O_6_: 421.1651; found: 421.1649. ^1^H NMR (500 MHz, DMSO-*d*6, Supporting Information (available here)): Table 1. ^13^C NMR (125 MHz, DMSO-*d*6, Supporting Information (available here)): Table 2.

#### 2.3.4. 3-(3,4-Dihydroxyphenyl)-10-((3,3-dimethyloxiran-2-yl)(methoxy)methyl)-5-hydroxy-8,8-dimethylpyrano[3,2-g]chromen-4(8H)-one (**IV**), Named Kazosajin IV

IR (neat) cm^−1^: 2926, 1632, 1584, 1435, 1248, 1138. HR-FTMS:* m*/*z* [M − H]^−^ calcd. for C_26_H_25_O_8_: 465.1549; found: 465.1466. ^1^H NMR (400 MHz, DMSO-*d*6, Supporting Information (available here)): Table 1. ^13^C NMR (100 MHz, DMSO-*d*6, Supporting Information (available here)): Table 2.

### 2.4. Cannabinoid and Opioid Receptor Assay

The affinities of the total extracts and the isolated compounds towards cannabinoid and opioid receptors were assessed according to the published method [20].

### 2.5. MAO-A and MAO-B Inhibition Assay

The inhibitory effects of the chemical components of* M. pomifera *(L.) on MAO-A and MAO-B were determined via the kynuramine deamination assay, where it was adapted for 96-well plates as described earlier [21, 22]. A fixed concentration of the substrate and varying concentrations of the inhibitor were used to determine the IC_50_ value at the point where 50% inhibition of the catalytic activity of the enzyme occurred. For MAO-A, the substrate (kynuramine) concentration of 80 *μ*M was chosen, since the *K*_*M*_ value of substrate binding reported previously was approximately 40 *μ*M [23]. *K*_*M*_ is the substrate concentration at half* V*max; therefore, 2 × *K*_*M*_ (2 × 40 = 80 *μ*M) was selected for determining the IC_50_ values. Similarly, for MAO-B, a substrate (kynuramine) concentration of 50 *μ*M was chosen. The assay was performed with the addition of the inhibitor. Inhibition was calculated as percent of the product formation compared to the corresponding control (enzyme-substrate reaction) without the inhibitors. The enzyme reactions were carried out in 0.1 M potassium phosphate buffer at pH 7.4. Reaction mixtures contained 5 *μ*g/mL of MAO-A (18.75 *μ*L in buffer) and 10 *μ*g/mL of MAO-B (18.75 *μ*L in buffer). The compounds were dissolved in DMSO and diluted in buffer. The total reaction mixture volume was 75 *μ*L, yielding a final DMSO concentration of 1.0% in the reaction mixture. The reaction mixtures were preincubated for 10 min at 37°C followed by the addition of MAO-A/MAO-B to initiate the reactions. The reaction mixtures were incubated for 20 min at 37°C and stopped by the addition of 28 *μ*L of 2 N NaOH. The formation of 4-hydroxyquinoline was determined fluorometrically by Spectra Max M5 fluorescence plate reader (Molecular Devices, Sunnyvale, CA, USA) with an excitation and emission wavelength of 320 nm and 380 nm, respectively, using the Soft Max Pro program [24]. Appropriate controls were set up to check the interference with the fluorescence measurements. None of the tested fractions or compounds showed any interference with the fluorescence measurement. The determination of IC_50_ values for inhibition of MAO-A and MAO-B by the* M. pomifera *(L.) compounds was performed using a fixed concentration of the substrate and varying the concentration of the inhibitor.* M. pomifera *compounds (0.01 *μ*M to 100 *μ*M) and clorgyline (0.001 *μ*M to 100 *μ*M) for MAO-A and deprenyl (0.001 *μ*M to 100 *μ*M) for MAO-B were tested to determine IC_50_ from the concentration dependent inhibition curves using XL-Fit^©^ software.

## 3. Results and Discussion

### 3.1. Structural Elucidation

Compound** I **was obtained as a yellow solid and exhibited a sodiated molecular ion peak in HR-FTMS at* m/z* 491.1690 corresponding to the molecular formula C_26_H_28_O_8_Na (that calculated for C_26_H_28_O_8_Na is 491.1682). ^1^H NMR (500 MHz, DMSO-*d*6) exhibited four methyl singlets at *δ* 1.43, 1.41, 1.21, and 1.13 which were assigned to H-4′′, 5′′, 4′′′, and 5′′′. A singlet at *δ* 3.19 was ascribed to the methoxy at C-3′′′. A doublet at *δ* 2.75 (5.4 Hz) was assigned to methylene protons H-1′′′. A multiplet integrating for one proton at *δ* 3.47 was assigned to an oxymethine proton at H-2′′′. Two doublets at *δ* 5.78 (10.0 Hz) and *δ* 6.63 (10.0 Hz) were assigned to H-2′′ and 1′′ methine protons. A singlet at *δ* 6.99 and multiplet at *δ* 6.76 were assigned to H-5′, H-2′, and H-6′. A singlet at *δ* 8.37 was assigned to oxygenated olefinic proton H-2.

The ^13^C NMR data of** I** (125 MHz, DMSO-*d*6) exhibited 26 carbon atoms. Four methyls at *δ* 27.9, 27.8, 21.8, and 20.6 were assigned to C-4′′, C-5′′, C-4′′′, and C-5′′′. Carbon at *δ* 48.6 is assigned to methoxy carbon at C-2′′′. Carbon at *δ* 74.0 was attributed to C-2′′′. Two oxygenated carbon atoms at *δ* 77.8 and 77.0 were assigned to 3′′ and 3′′′. The peaks at *δ* 156.6, 155.1, 154.3, 154.1, 145.6, and 144.9 were ascribed to C-5, C-7, C-2, C-8a, C-3′, and C-4′. The peak at *δ* 181.0 was assigned to the carbonyl carbon at C-4. The HMBC spectrum of** I** showed key ^3^*J* and ^2^*J* correlations between the methoxy proton at *δ* 3.47 and the oxygenated methine carbon at C-2′′′, confirming the position of the methoxy at C-2′′′. COSY correlations have been noticed between H-1′′ to H-2′′ and H-1′′′ to H-2′′′. Hence, the structure of compound** I** is deduced to be 3-(3,4-dihydroxyphenyl)-5-hydroxy-10-(3-hydroxy-2-methoxy-3-methylbutyl)-8,8-dimethylpyrano[3,2-g]chromen-4(8H)-one (**I**) and named Kazosajin** I**.

Compound** II **was obtained as a yellow solid and exhibited a peak in HR-FTMS at* m/z* 451.1415 [M – H]^−^ corresponding to the molecular formula C_25_H_23_O_8_ (that calculated for C_25_H_23_O_8_ is 451.1393). The ^1^H NMR (500 M Hz, CDCl_3_) showed singlets at *δ* 1.46 integrating for six protons and at *δ* 1.74 integrating for three protons, confirming the presence of three methyls at C-4′′′, C-5′′′, and C-5′′. A downfield singlet at *δ* 4.52 (s) was ascribed to the H-2′′. Two singlets at *δ* 4.77 and 4.67 were assigned to exomethylene protons H-4′′. Two doublets at *δ* 6.66 (10.0 Hz) and 5.77 (10.0 Hz) were assigned to the two olefinic protons at H-1′′′ and H-2′′′. Two doublets at *δ* 6.93 (1.8 Hz) and 6.79 (1.8 Hz) and a singlet at *δ* 7.00 were assigned to trisubstituted benzene ring protons at H-6′, H-5′, and H-2′. An oxygenated olefinic proton H-2 appeared as a singlet at *δ* 8.31. ^13^C NMR (125 MHz, CDCl_3_) of** II** exhibited 26 carbon atoms. It showed three methyls at *δ* 27.9, 27.7, and 16.7 which were attributed to C-4′′′, C-5′′′, and C-5′′. Methine carbon at *δ* 86.7 and quaternary carbon at 78.3 were assigned to C-2′′ and C-3′′′. The peaks at *δ* 159.3, 156.8, 154.0, 150.8, 145.6, and 144.9 were ascribed to the oxygenated carbon atoms at C-5, C-7, C-2, C-8a, C-3′, and C-4′. The peaks at *δ* 195.4 and 181.2 were ascribed to carbonyls at C-1′′ and C-3. The HMBC spectrum of** II** showed ^3^*J* and ^2^*J* correlations between the H-5′′ and C-2′′, C-3′′, and C-4′′, and H-2′′ with C-1′′ indicated the presence of a prenylated side chain with *α*-hydroxy ketone. Hence, the structure of compound** II** is deduced to be 3-(3,4-dihydroxyphenyl)-5-hydroxy-6-(2-hydroxy-3-methylbut-3-enoyl)-8,8-dimethylpyrano[2,3-f]chromen-4(8H)-one (**II**) and named Kazosajin** II**.

Compound** III **was obtained as a yellow solid and exhibited a molecular ion peak in HR-FTMS at* m/z* 421.1649 corresponding to the molecular formula C_25_H_25_O_6_ (that calculated for C_25_H_25_O_6_ is 421.1651). The spectral data of** III** is similar to that of iso-osajin except for the hydroxylation at C-2′′. ^1^H NMR (400 M Hz, DMSO-*d*6) showed five singlets at *δ* 1.45, 1.44, 1.30, and 1.20 assigned to H-4′′, H-5′′, H-4′′′, and H-5′′′. Two resonances at *δ* 2.79 (dd, 5.3, 17.0 Hz) and *δ* 2.43 (dd, 7.3, 17.0 Hz) are attributed to H_2_-1′′. The triplet at *δ* 3.66 (6.3 Hz) was ascribed to oxymethine proton at H-2′′. The two doublets at *δ* 5.73 (9.9 Hz) and 6.68 (9.9 Hz) are assigned to the olefinic protons H-1′′′ and H-2′′′. The two doublets at *δ* 6.79 (8.4) and 7.30 (8.4) were ascribed to four aromatic protons H-2′, H-3′, H-5′, and H-6′ on a* p*-disubstituted benzene ring. A singlet at 8.10 was assigned to oxygenated olefinic proton H-2.^13^C NMR (100 MHz, DMSO-*d*6) of** III** exhibited 25 carbon atoms. It showed four methyls at 27.9, 27.8, 25.4, and 20.7 attributed to C-4′′′, C-5′′′, C-4′′, and C-5′′. It showed three sp^3^ oxygenated carbon atoms at *δ* 78.1, 77.8, and 66.6 which were assigned to C-3′′, C-3′′′, and C-2′′, respectively. It exhibited five oxygenated aromatic carbon atoms at *δ* 157.1, 154.3, 153.8, 151.8, and 150.1 which were assigned to C-4′, C-5, C-8a, C-7, and C-2. The peak at *δ* 173.8 was ascribed to the carbonyl carbon at C-3. The HMBC spectrum of** III** showed ^3^*J* and ^2^*J* correlations between the methyl protons at H-4′′, 5′′ to C-2′′, and 3′′ indicating the presence of a hydroxyl group at C-2′′. Hence, the structure of compound** III** is deduced to be 11-hydroxy-7-(4-hydroxyphenyl)-2,2,10,10-tetramethyl-11,12-dihydro-2H-dipyrano[2,3-f: 2′,3′-h]chromen-8(10H)-one (**III**) and named Kazosajin** III**.

Compound** IV **was obtained as a yellow solid and exhibited a peak in HR-FTMS at* m/z* 465.1466 [M − H]^−^ corresponding to the molecular formula C_26_H_26_O_8_ (that calculated for C_26_H_25_O_8_ is 465.1549). ^1^H NMR (400 M Hz, CDCl_3_) showed four singlets at *δ* 1.44, 1.43, 1.19, and 1.06 for the presence of four methyls at C-4′′, C-5′′, C-4′′′, and C-5′′′. A downfield singlet at *δ* 3.22 was ascribed to the methoxy protons at C-1′′′. Two doublets at *δ* 4.54 (7.3 Hz) and 3.65 (7.3 Hz) were assigned to oxymethine protons H-1′′′ and H-2′′′ and the two doublets at *δ* 6.63 (10.0 Hz) and 5.82 (10.0 Hz) were assigned to two olefinic protons at H-1′′ and H-2′′. A singlet at *δ* 6.81 and two doublets at *δ* 7.03 (1.9 Hz) and 6.80 (1.9 Hz) were assigned to the three aromatic protons at H-2′, H-5′, and H-6′. An oxygenated olefinic proton H-2 appeared as a singlet at *δ* 8.42. ^13^C NMR (100 MHz, CDCl_3_) of** IV** exhibited 26 carbon atoms. It showed four methyls at *δ* 28.4, 28.1, 25.0, and 19.6 attributed to C-4′′, C-5′′, C-4′′′, and C-5′′′. Downfield methyl carbon at *δ* 56.4 was assigned to the methoxy carbon at C-1′′′. The four sp^3^ oxygenated carbon atoms at *δ* 75.0, 65.0, 79.0, and 57.3 were assigned to C-1′′′, C-2′′′, C-3′′, and C-3′′′. The peaks at *δ* 157.3, 156.4, 155.3, 154.5, 146.2, and 145.4 were ascribed to the oxygenated sp^2^ carbon atoms at C-5, C-7, C-8a, C-2, C-3′, and C-4′. The peak at *δ* 181.2 was ascribed to the carbonyl at C-3. The HMBC spectrum of** IV** showed ^3^*J* and ^2^*J* correlations between the methyl protons at H-4′′′ and 5′′′ to C-2′′′ and 3′′′ indicating the presence of an epoxy system between C-2′′′ and C-3′′′ which was further confirmed by HRMS. Hence, the structure of compound** IV** is deduced to be 3-(3,4-dihydroxyphenyl)-10-((3,3-dimethyloxiran-2-yl)(methoxy) methyl)-5-hydroxy-8,8-dimethylpyrano[3,2-g]chromen-4(8H)-one (**IV**) and named Kazosajin** IV**.

Compounds** V**–**XV** were isolated and identified by comparing their NMR data with the literature to be pomiferin (**V**) [16], auriculasin (**VI**) [16], warangalone (**VII**) [17], osajin (**VIII**) [16], artocarpesin (**IX**) [16], tonkinensisol (**X**) [18], corchoionoside B (**XI**) [19], kaempferol-7-*O*-*β*-*D*-glucoside (**XII**) [16], dihydrokaempferol-7-*O*-*β*-*D*-glucoside (**XIII**) [16], stigmasterol (**XIV**), and *β*-sitosterol (**XV**).

### 3.2. Cannabinoid and Opioid Receptors Assay

The affinities of the total extracts and the isolated compounds towards cannabinoid and opioid receptors were assessed using CP-55940 and Naloxone as controls for cannabinoid and opioid receptors assays, respectively. The total extract showed significant activity towards cannabinoid receptor (CB1: 103.4% displacement; CB2: 68.8% displacement), possibly allosteric towards *δ* and *μ* opioid receptors (−49.7 and −53.8% displacement, resp.). The new compound Kazosajin** I** was found to be possibly an allosteric compound in *κ* and *μ* opioid receptors (−88.4 and −27.2% displacement, resp.). Kazosajin** II** exhibited moderate activity towards cannabinoid receptors (CB1: 47.9% displacement; CB2: 42.3% displacement). The known compounds—pomiferin (**V**) (CB1: 107.2% displacement; CB2: 66.0% displacement), osajin (**VIII**) (CB1: 93.9% displacement; CB2: 52.7% displacement), and warangalone (**VII**) (CB1: 111.3% displacement; CB2: 77.57% displacement)—exhibited prominent activity towards cannabinoid receptors. The IC_50_ and* Ki *values for CB1 and CB2 receptors active compounds are given in Table 3.

### 3.3. Determination of the Inhibitory Effect of* M. pomifera* (L.) Compounds on Recombinant Human MAO-A and MAO-B

The compounds isolated from* M. pomifera *(L.) fruits were tested for their inhibitory effect against recombinant human MAO isoforms (MAO-A and MAO-B)* in vitro*. The enzymatic activity of MAO-A and MAO -B was determined via a fluorescence based method [24]. All the tested compounds showed fewer inhibitory effects on MAO-A compared to MAO-B activities (Table 4).

## 4. Conclusions

The* Maclura pomifera* total extract of the fruits showed significant activity towards cannabinoid receptors and possibly allosteric interactions with *δ* and *μ* opioid receptors [25]. Four new compounds (**I**–**IV**) along with eleven known compounds (**V**–**XV**) were isolated and identified from the extract. The new compound Kazosajin** I** was found to be possibly allosteric towards *κ* and *μ* opioid receptors, while the new compound Kazosajin** II** exhibited moderate activity towards cannabinoid receptors CB1 and CB2. Compounds** V**,** VII**, and** VIII** exhibited prominent activity towards cannabinoid receptors. All the isolated compounds from* M. pomifera *(L.) fruits were tested for their inhibitory effect against recombinant human MAO isoforms (MAO-A and MAO-B)* in vitro*, where four compounds (**II**,** III**,** VI**, and** VII**) showed selective inhibition of MAO-B. All the tested compounds showed fewer inhibitory effects on MAO-A compared to MAO-B activities.

## Figures and Tables

**Table 1:** ^ 1^H NMR data of compounds **I**–**IV**.

Proton	^a^Compound **I**	^a^Compound **II**	^a^Compound **III**	^b^Compound **IV**
2	8.37 (s)	8.31 (s)	8.10 (s)	8.42 (s)
2′	6.76 (m)	7.00 (s)	6.79 (d, 8.4)	6.81 (s)
3′	-	-	7.30 (d, 8.4)	-
5′	6.99 (s)	6.93 (d, 1.8)	7.30 (d, 8.4)	7.03 (d, 1.9)
6′	6.76 (m)	6.79 (d, 1.8)	6.79 (d, 8.4)	6.80 (d, 1.9)
1′′	6.63 (d, 10.0)	-	2.79 (dd, 5.6, 17.0), 2.43 (dd, 5.6, 17.0)	6.63 (d, 10.0)
2′′	5.78 (d, 10.0)	4.52 (s)	3.66 (dd, 5.6, 7.1)	5.82 (d, 10.0)
4′′	1.43 (s)	4.77 (s), 4.67 (s)	1.30 (s)	1.44 (s)
5′′	1.41 (s)	1.74 (s)	1.20 (s)	1.43 (s)
1′′′	2.75 (d, 5.4)	5.77 (d, 10.0)	5.73 (d, 9.9)	4.54 (d, 7.3)
2′′′	3.47 (m)	6.66 (d, 10.0)	6.68 (d, 9.9)	3.65 (d, 7.3)
4′′′	1.13 (s)	1.46 (s)	1.45 (s)	1.19 (s)
5′′′	1.21 (s)	1.46 (s)	1.44 (s)	1.06 (s)
OMe	3.19 (s)	-	-	3.22 (s)

^a^Data acquired at 500 MHz. ^b^Data acquired at 400 MHz.

**Table 2:** ^13^C NMR data of compounds **I**–**IV**.

Carbon	^a^Compound **I**	^a^Compound **II**	^a^Compound **III**	^b^Compound **IV**
2	154.3	154.0	150.1	154.5
3	122.1	122.6	124.8	123.0
4	181.0	180.6	173.8	181.2
4a	105.2	104.6	105.3	105.8
5	156.6	159.3	154.3	157.3
6	104.4	108.1	108.3	104.9
7	155.1	156.8	151.8	156.4
8	106.4	100.0	100.8	104.3
8a	154.1	150.1	153.8	155.3
1′	121.6	121.5	122.7	121.7
2′	119.9	120.0	114.8	120.4
3′	145.6	145.6	130.4	146.2
4′	144.9	144.9	157.1	145.4
5′	116.6	116.6	130.4	117.0
6′	115.5	115.4	114.8	115.9
1′′	115.2	195.4	25.7	115.1
2′′	128.7	86.7	66.6	129.4
3′′	77.8	144.6	78.1	79.0
4′′	27. 9	112.9	25.4	28.4
5′′	27.8	16.7	20.7	28.1
1′′′	24.3	127.7	127. 6	75.0
2′′′	74.0	114.1	114. 7	65.0
3′′′	77.0	78.3	77.8	57.3
4′′′	21.8	27.8	27.9	25.0
5′′′	20.6	27.7	27.8	19.6
OMe	48.6	-	-	56.4

^a^Data acquired at 125 MHz. ^b^Data acquired at 100 MHz.

**Table 3:** Cannabinoid receptors activity of potential constituents (10 *μ*M) from* M. pomifera*.

Compound	CB1			CB2		
	% displacement	IC_50_ (*μ*M)	*Ki* (*μ*M)	% displacement	IC_50_ (*μ*M)	*Ki *(*μ*M)
Pomiferin (**V**)	107.2	2.110	1.055	66.0	1.318	6.590
Auriculasin (**VI**)	86.7	8.923	4.462	-	-	-
Warangalone (**VII**)	111.3	1.670	8.350	77.6	4.438	2.219
Osajin (**VIII**)	93.9	3.859	1.929	52.7	7.646	3.823

**Table 4:** Inhibition of recombinant human monoamine oxidase-A and monoamine oxidase-B of **I**–**VIII **from *M. pomifera*.

Compounds	Monoamine oxidase-A IC_50_ (*μ*M)	Monoamine oxidase-B IC_50_ (*μ*M)	SI index MAO-A/B
Kazosajin **I **	74.33 ± 3.44	11.59 ± 1.39	6.413
Kazosajin **II **	72.03 ± 3.72	4.28 ± 0.67	16.829
Kazosajin **III **	>100	7.16 ± 1.15	-
Kazosajin **IV **	71.20 ± 2.61	17.66 ± 1.06	4.032
Pomiferin (**V**)	>100	>100	-
Auriculasin (**VI**)	45.98 ± 4.48	1.91 ± 0.32	24.073
Warangalone (**VII**)	>100	5.69 ± 0.38	-
Osajin (**VIII**)	>100	>100	-
Clorgyline	0.0045 ± 0.0004	-	-
Deprenyl	-	0.0326 ± 0.012	-

*Notes*. The results of IC_50_ values are expressed as mean ±SD of triplicate observations.

## Supplementary Materials

Supplementary Material 1D and 2D NMR and mass data for compounds** I**–**IV**; this material is available free of charge via the Internet through the journal's website. Figure S1: ^1^H NMR spectrum for compound** I** (DMSO-*d*6, 500 MHz). Figure S2: ^13^C NMR spectrum for compound** I** (DMSO-*d*6, 125 MHz). Figure S3: DEPT spectrum for compound** I** (DMSO-*d*6, 125 MHz). Figure S4: HSQC spectrum for compound** I** (DMSO-*d*6, 500 MHz). Figure S5: HMBC spectrum for compound** I** (DMSO-*d*6, 500 MHz). Figure S6: COSY spectrum for compound** I** (DMSO-*d*6, 500 MHz). Figure S7: FTMS spectrum for compound** I**. Figure S8: ^1^H NMR spectrum for compound** II** (DMSO-*d*6, 500 MHz). Figure S9: ^13^C NMR spectrum for compound** II** (DMSO-*d*6, 125 MHz). Figure S10: DEPT spectrum for compound** II** (DMSO-*d*6, 125 MHz). Figure S11: HSQC spectrum for compound** II** (DMSO-*d*6, 500 MHz). Figure S12: HMBC spectrum for compound** II** (DMSO-*d*6, 500 MHz). Figure S13: FTMS spectrum for compound** II**. Figure S14: ^1^H NMR spectrum for compound** III** (DMSO-*d*6, 500 MHz). Figure S15: ^13^C NMR spectrum for compound** III** (DMSO-*d*6, 125 MHz). Figure S17: HSQC spectrum for compound** III** (DMSO-*d*6, 500 MHz). Figure S18: HMBC spectrum for compound** III** (DMSO-*d*6, 500 MHz). Figure S19: FTMS spectrum for compound** III**. Figure S20: ^1^H NMR spectrum for compound** IV** (DMSO-*d*6, 400 MHz). Figure S21: ^13^C NMR spectrum for compound** IV** (DMSO-*d*6, 100 MHz). Figure S22: DEPT spectrum for compound** IV** (DMSO-*d*6, 100 MHz). Figure S23: HSQC spectrum for compound** IV** (DMSO-*d*6, 400 MHz). Figure S24: HMBC spectrum for compound** IV** (DMSO-*d*6, 400 MHz). Figure S25: COSY spectrum for compound** IV** (DMSO-*d*6, 400 MHz). Figure S26: NOESY spectrum for compound** IV** (DMSO-*d*6, 400 MHz). Figure S27: FTMS spectrum for compound** IV**.
