# Supplementary material for: Isolation and Biological Evaluation of Prenylated Flavonoids from Maclura pomifera
Source: Evid Based Complement Alternat Med. 2018 Jan 14;2018:1370368. doi: 10.1155/2018/1370368 (PMC5820588; doi:10.1155/2018/1370368)
Supplement: Supplementary Material — 1D and 2D NMR and mass data for compounds I–IV; this material is available free of charge via the Internet through the journal's website. Figure S1: 1H NMR spectrum for compound I (DMSO-d6, 500 MHz). Figure S2: 13C NMR spectrum for compound I (DMSO-d6, 125 MHz). Figure S3: DEPT spectrum for compound I (DMSO-d6, 125 MHz). Figure S4: HSQC spectrum for compound I (DMSO-d6, 500 MHz). Figure S5: HMBC spectrum for compound I (DMSO-d6, 500 MHz). Figure S6: COSY spectrum for compound I (DMSO-d6, 500 MHz). Figure S7: FTMS spectrum for compound I. Figure S8: 1H NMR spectrum for compound II (DMSO-d6, 500 MHz). Figure S9: 13C NMR spectrum for compound II (DMSO-d6, 125 MHz). Figure S10: DEPT spectrum for compound II (DMSO-d6, 125 MHz). Figure S11: HSQC spectrum for compound II (DMSO-d6, 500 MHz). Figure S12: HMBC spectrum for compound II (DMSO-d6, 500 MHz). Figure S13: FTMS spectrum for compound II. Figure S14: 1H NMR spectrum for compound III (DMSO-d6, 500 MHz). Figure S15: 13C NMR spectrum for compound III (DMSO-d6, 125 MHz). Figure S17: HSQC spectrum for compound III (DMSO-d6, 500 MHz). Figure S18: HMBC spectrum for compound III (DMSO-d6, 500 MHz). Figure S19: FTMS spectrum for compound III. Figure S20: 1H NMR spectrum for compound IV (DMSO-d6, 400 MHz). Figure S21: 13C NMR spectrum for compound IV (DMSO-d6, 100 MHz). Figure S22: DEPT spectrum for compound IV (DMSO-d6, 100 MHz). Figure S23: HSQC spectrum for compound IV (DMSO-d6, 400 MHz). Figure S24: HMBC spectrum for compound IV (DMSO-d6, 400 MHz). Figure S25: COSY spectrum for compound IV (DMSO-d6, 400 MHz). Figure S26: NOESY spectrum for compound IV (DMSO-d6, 400 MHz). Figure S27: FTMS spectrum for compound IV. [file 1370368.f1.pdf]

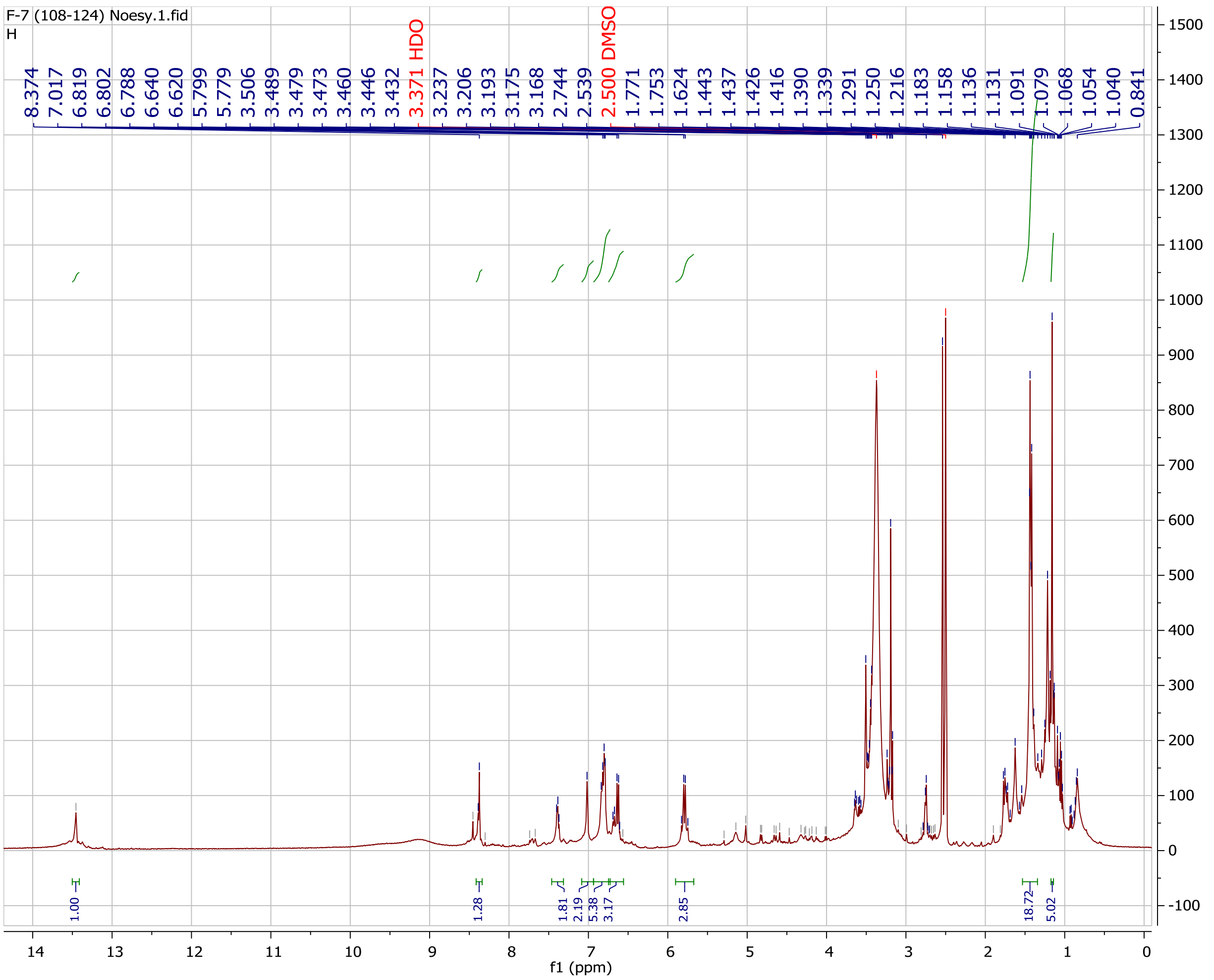

Figure S1: ^{1}H NMR spectrum for compound **I** (DMSO-*d*6, 500 MHz)

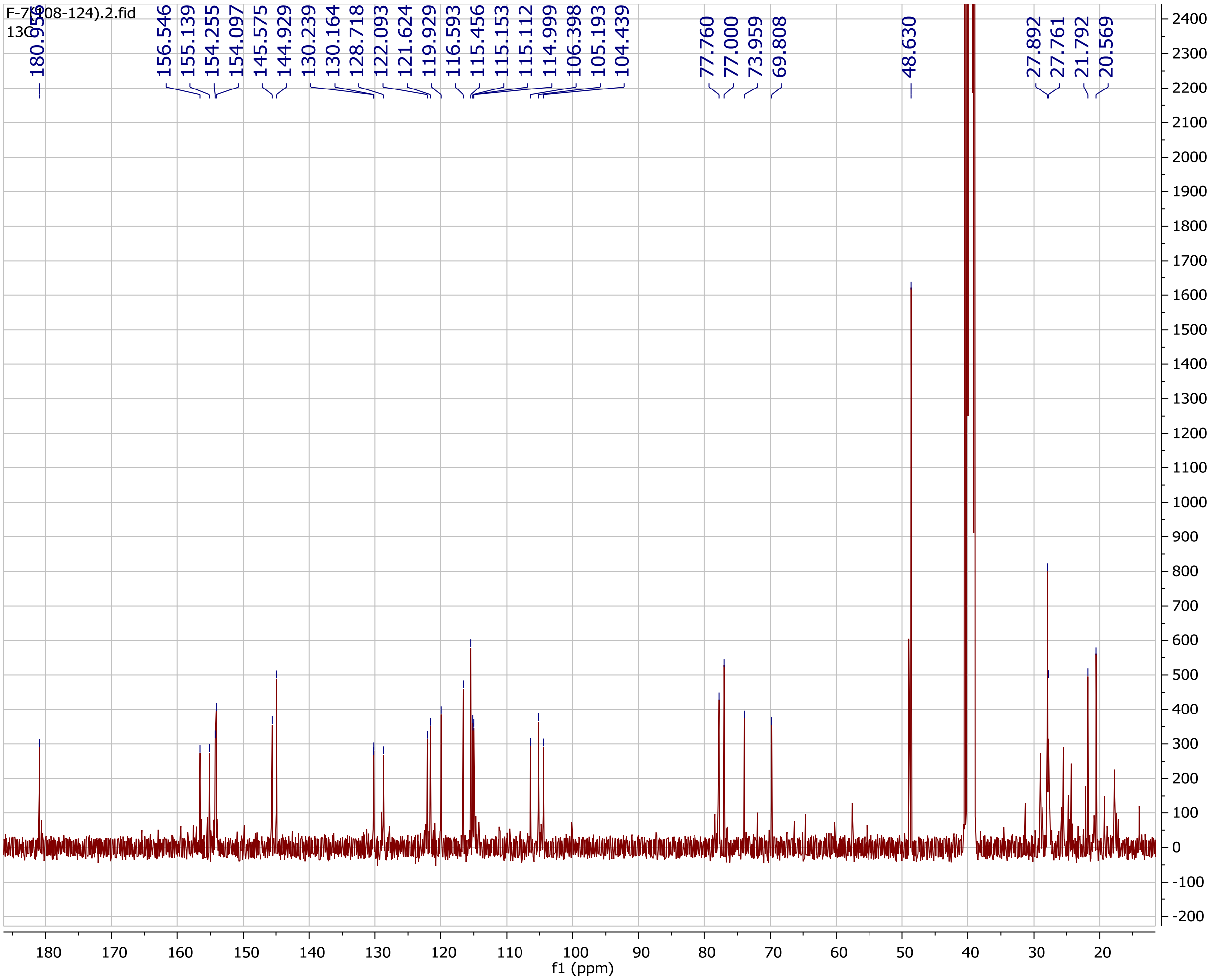

Figure S2: ^{13}C NMR spectrum for compound **I (**DMSO-*d*6, 125 MHz)

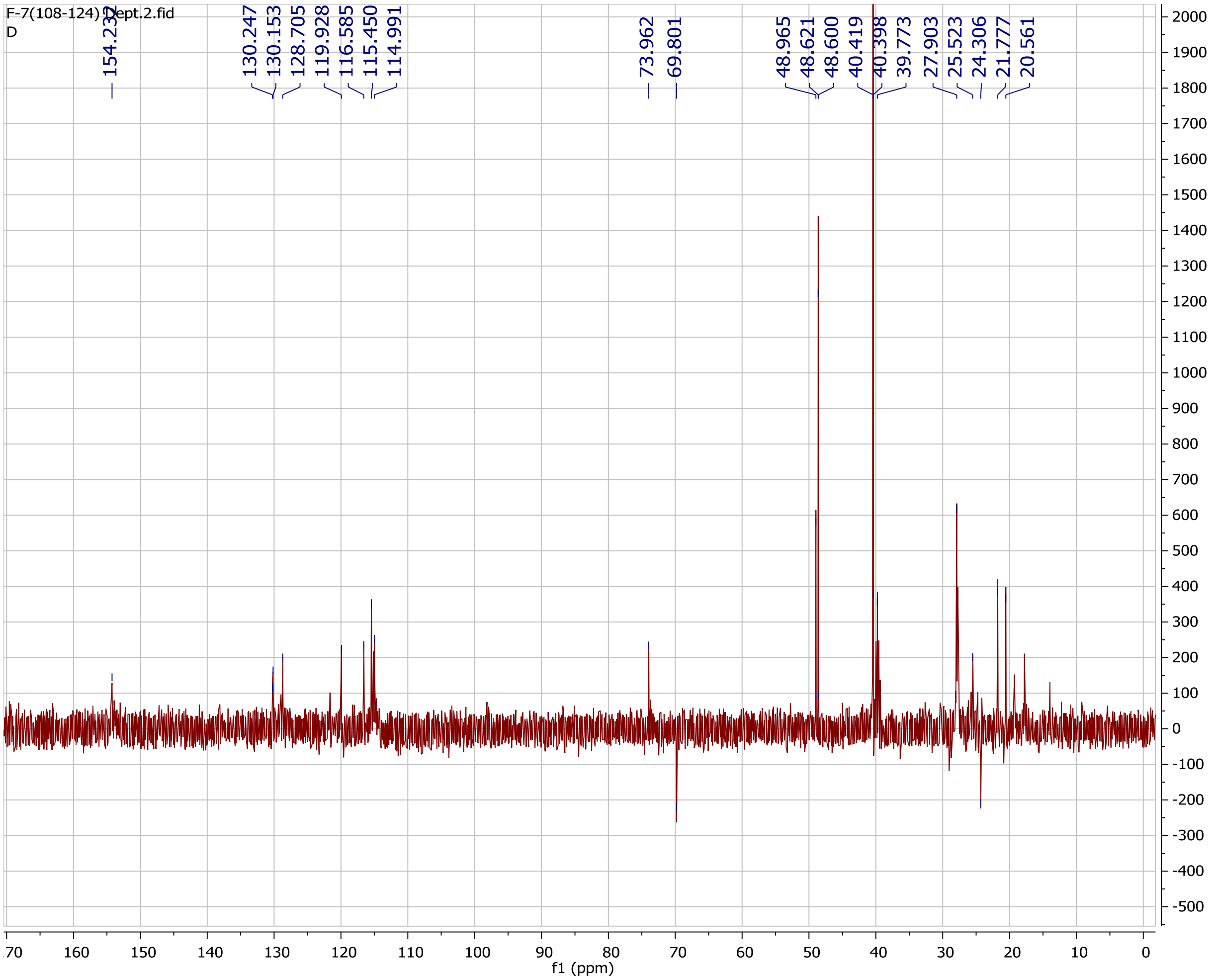

Figure S3: DEPT spectrum for compound **I (**DMSO-*d*6, 125 MHz)

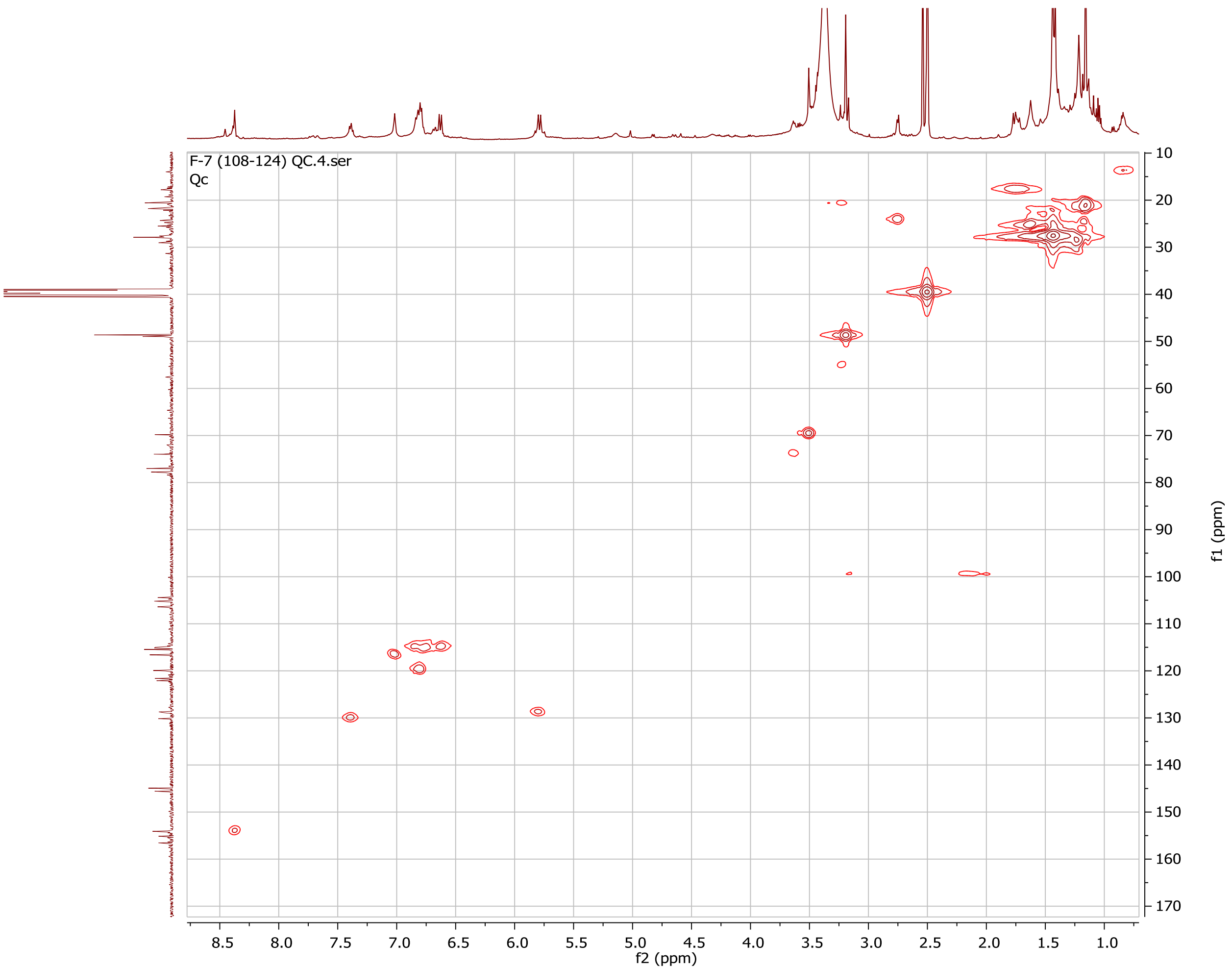

Figure S4: HSQC spectrum for compound **I** (DMSO-*d*6, 500 MHz)

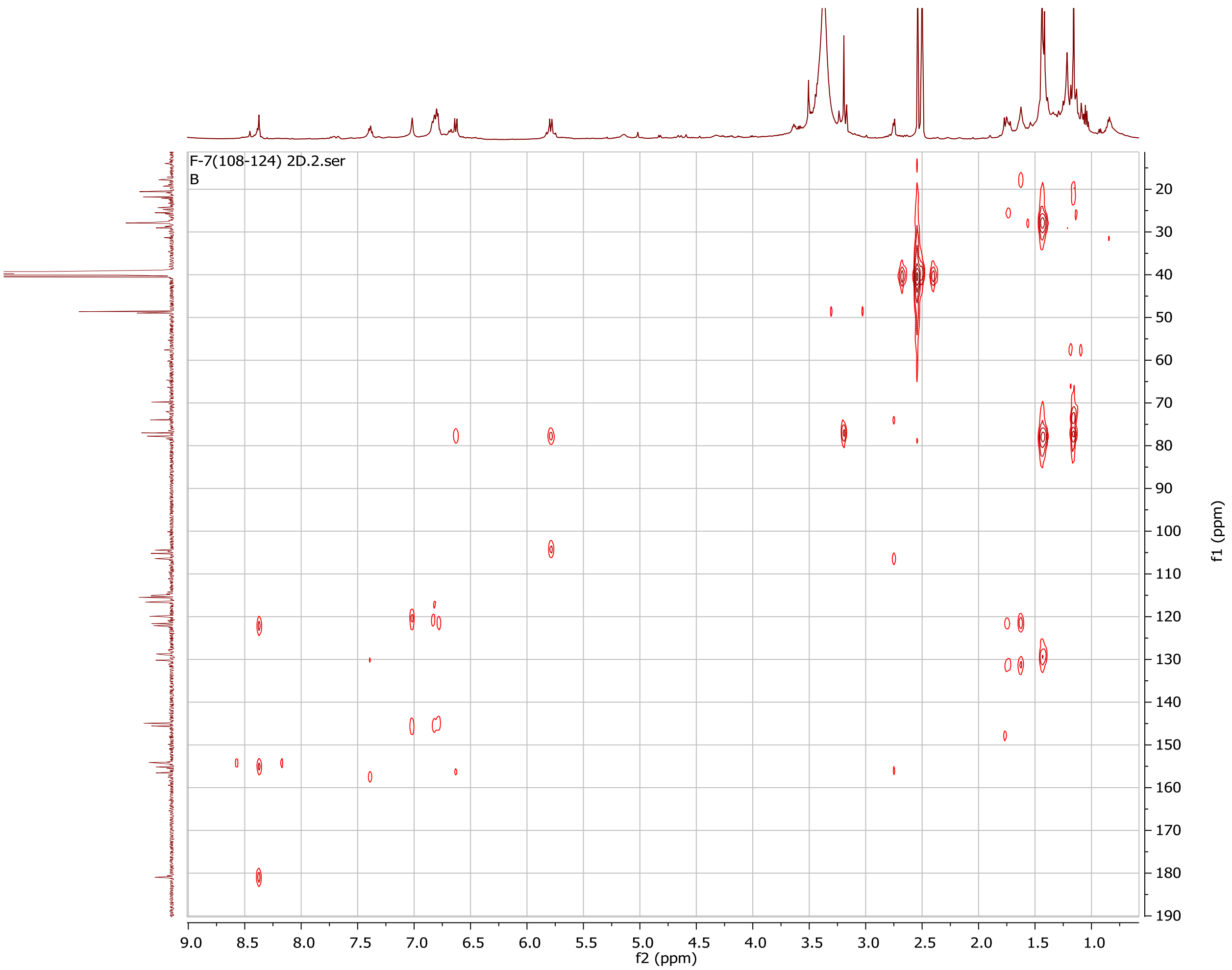

Figure S5: HMBC spectrum for compound **I** (DMSO-*d*6, 500 MHz)

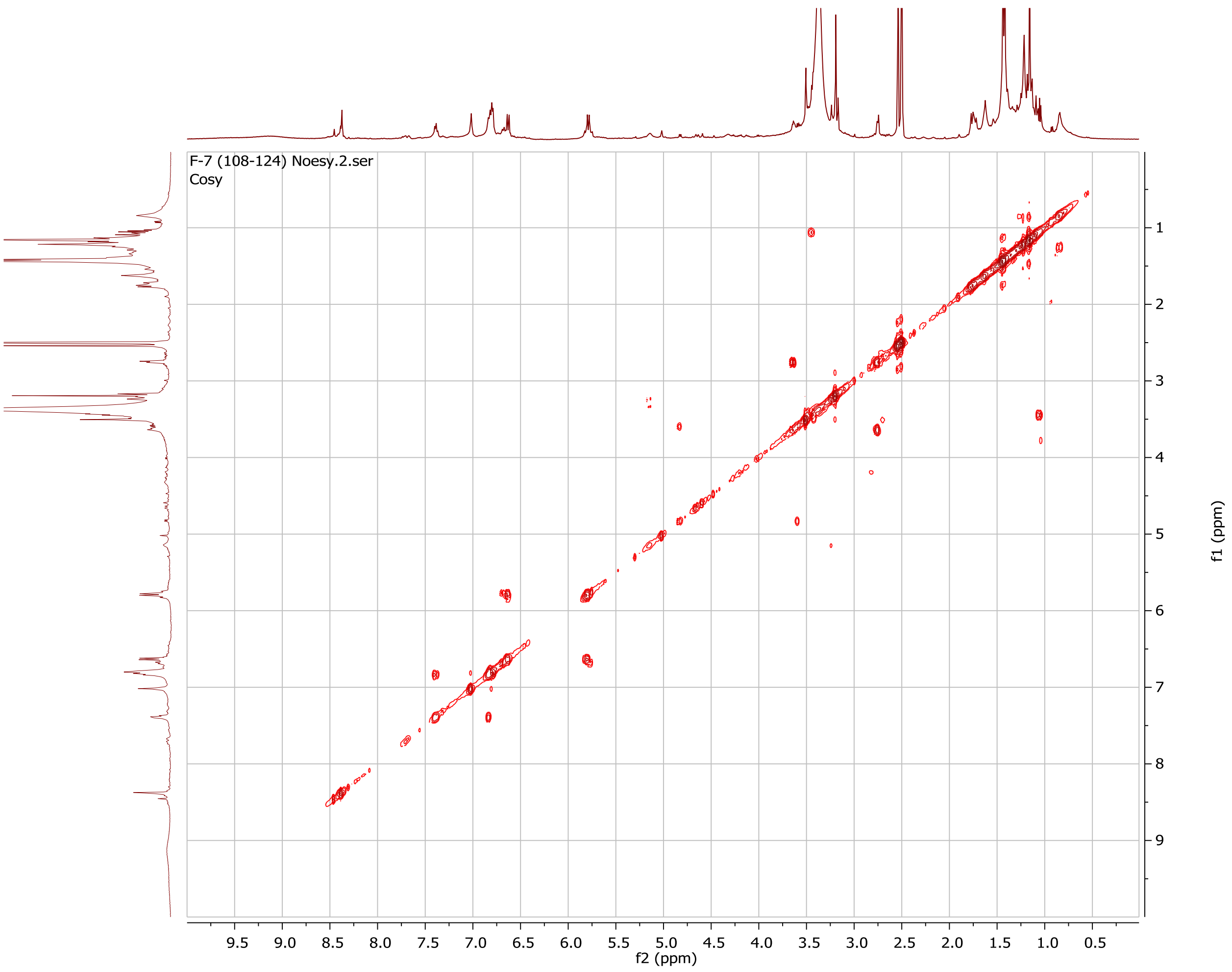

Figure S6: COSY spectrum for compound **I** (DMSO-*d*6, 500 MHz)

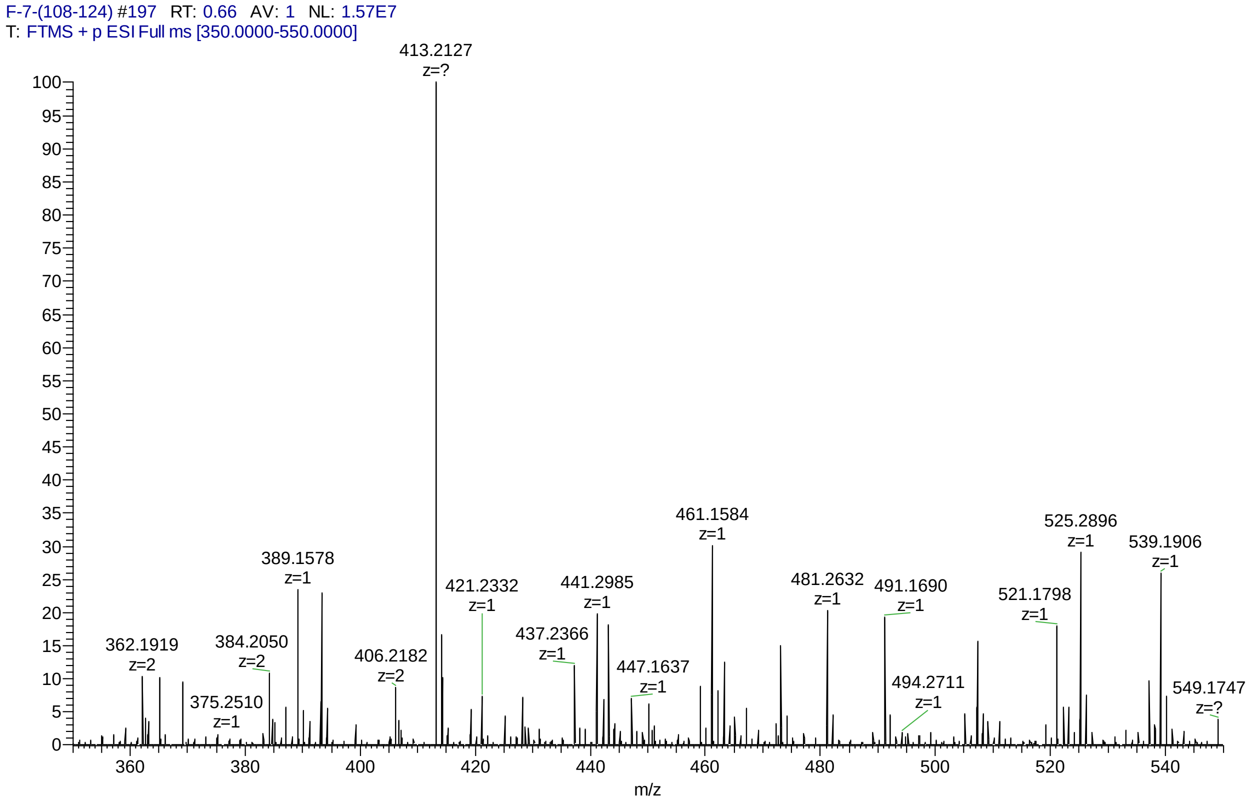

Figure S7: FT-MS spectrum for compound **I**

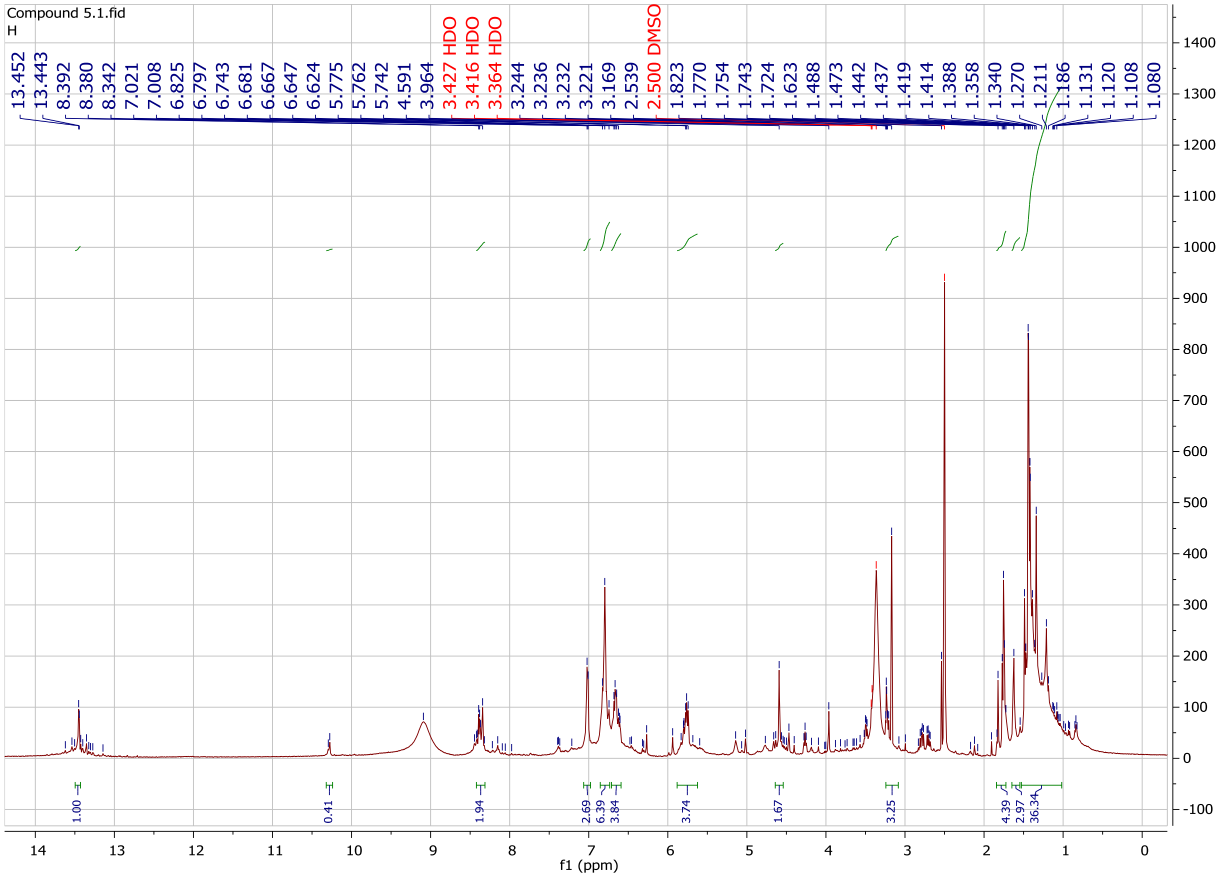

Figure S8: ^{1}H NMR spectrum for compound **II** (DMSO-*d*6, 500 MHz)

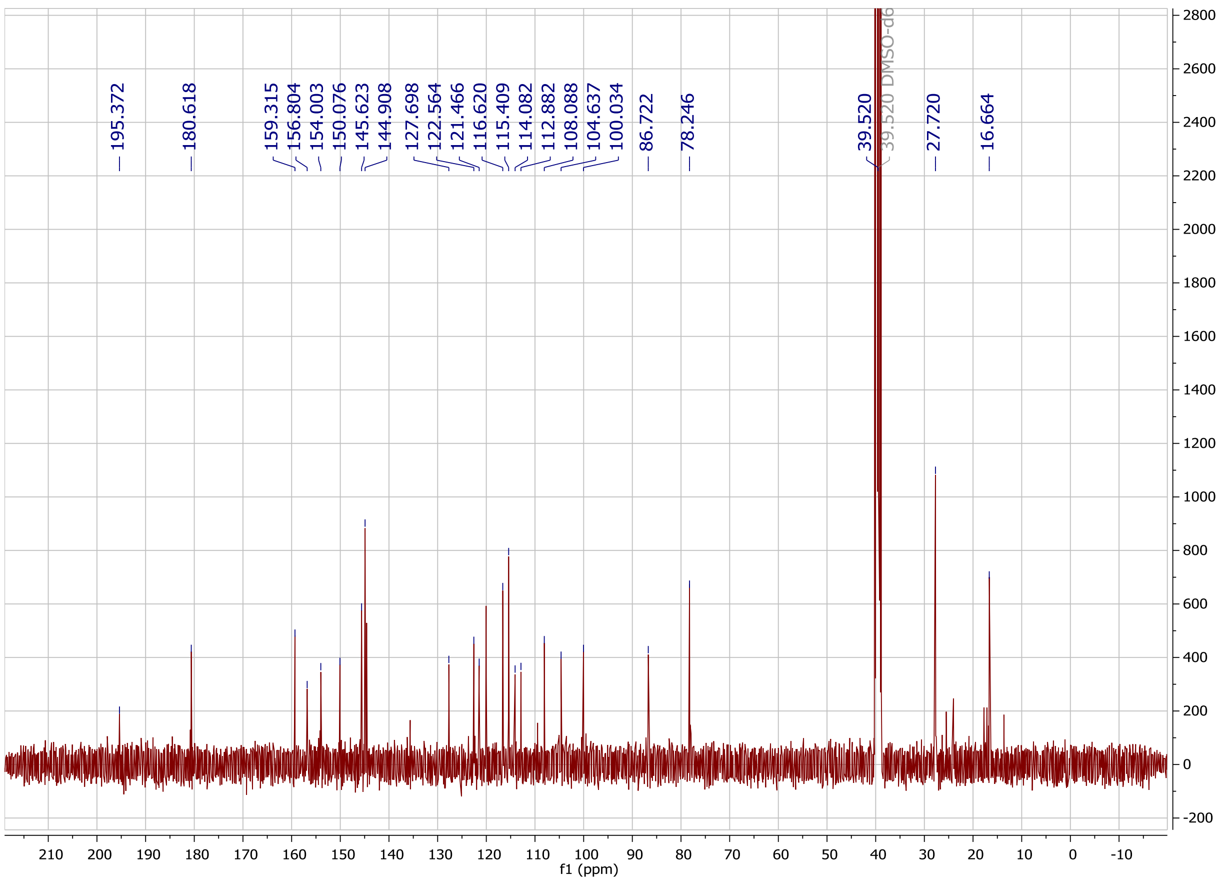

Figure S9: ^{13}C NMR spectrum for compound **II (**DMSO-*d*6, 125 MHz)

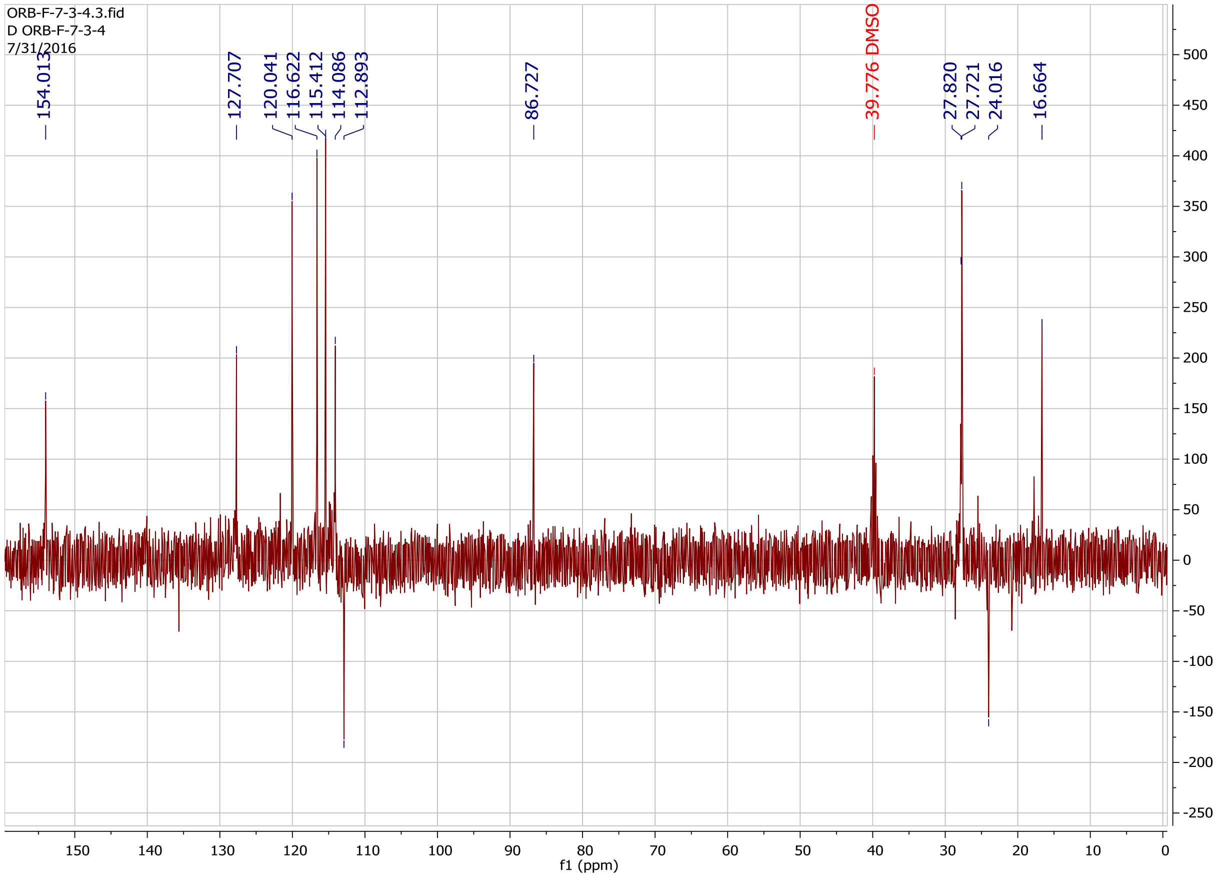

Figure S10: DEPT spectrum for compound **II (**DMSO-*d*6, 125 MHz)

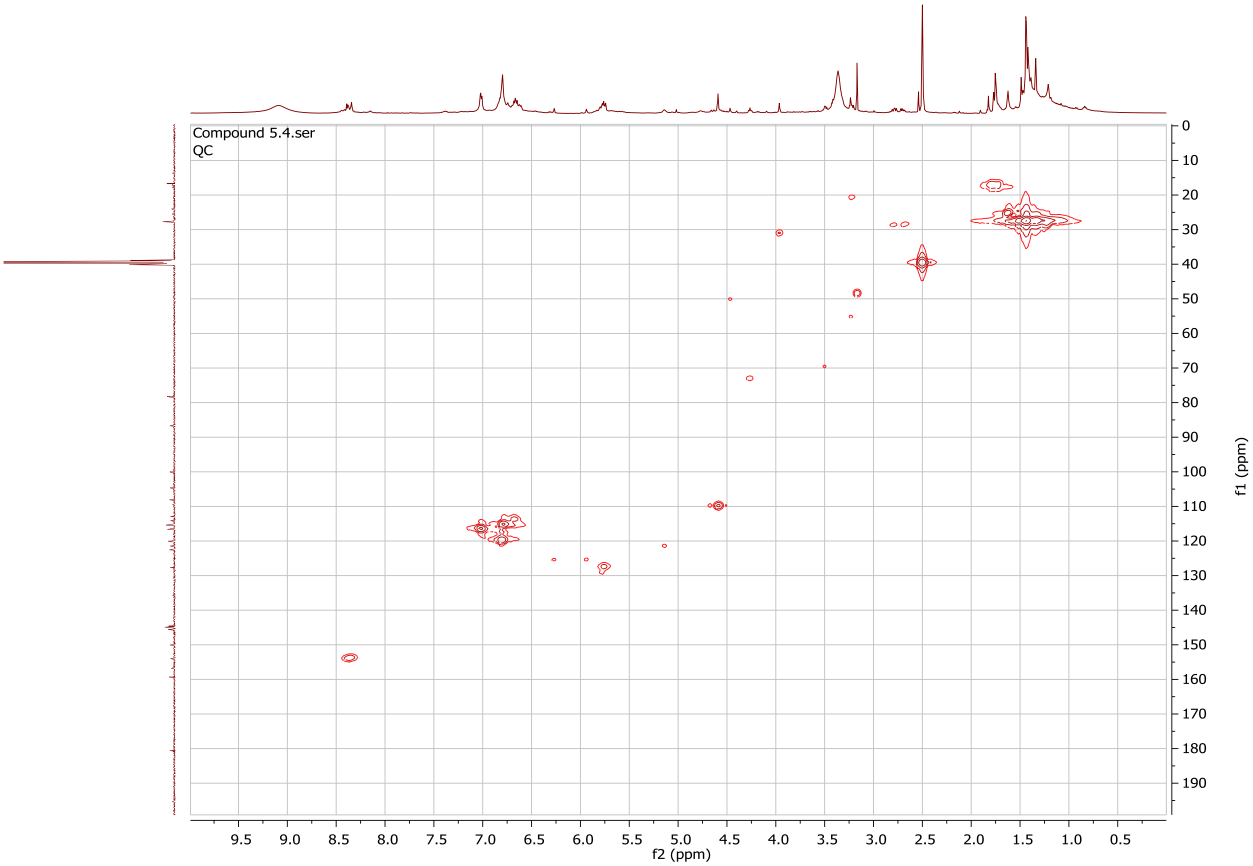

Figure S11: HSQC spectrum for compound **II** (DMSO-*d*6, 500 MHz)

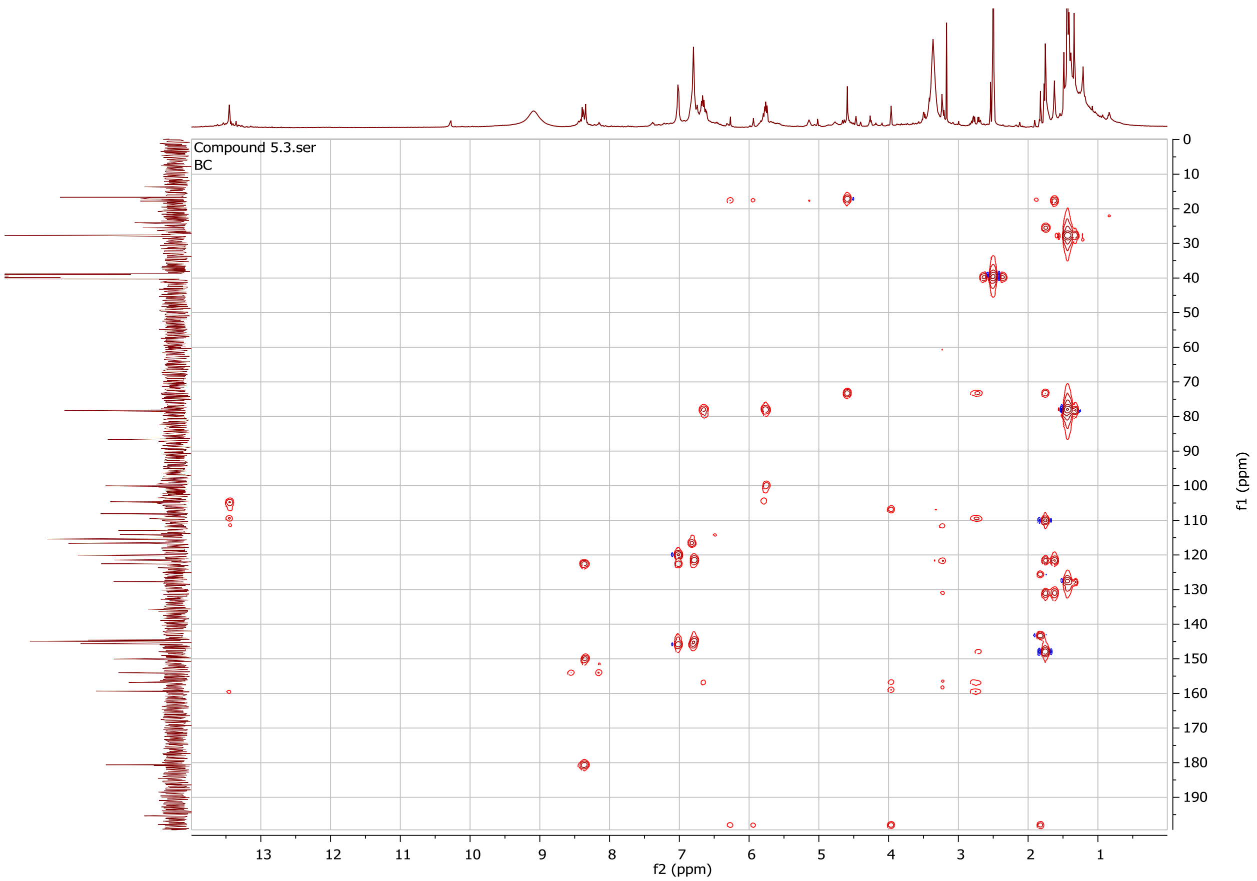

Figure S12: HMBC spectrum for compound **II** (DMSO-*d*6, 500 MHz)

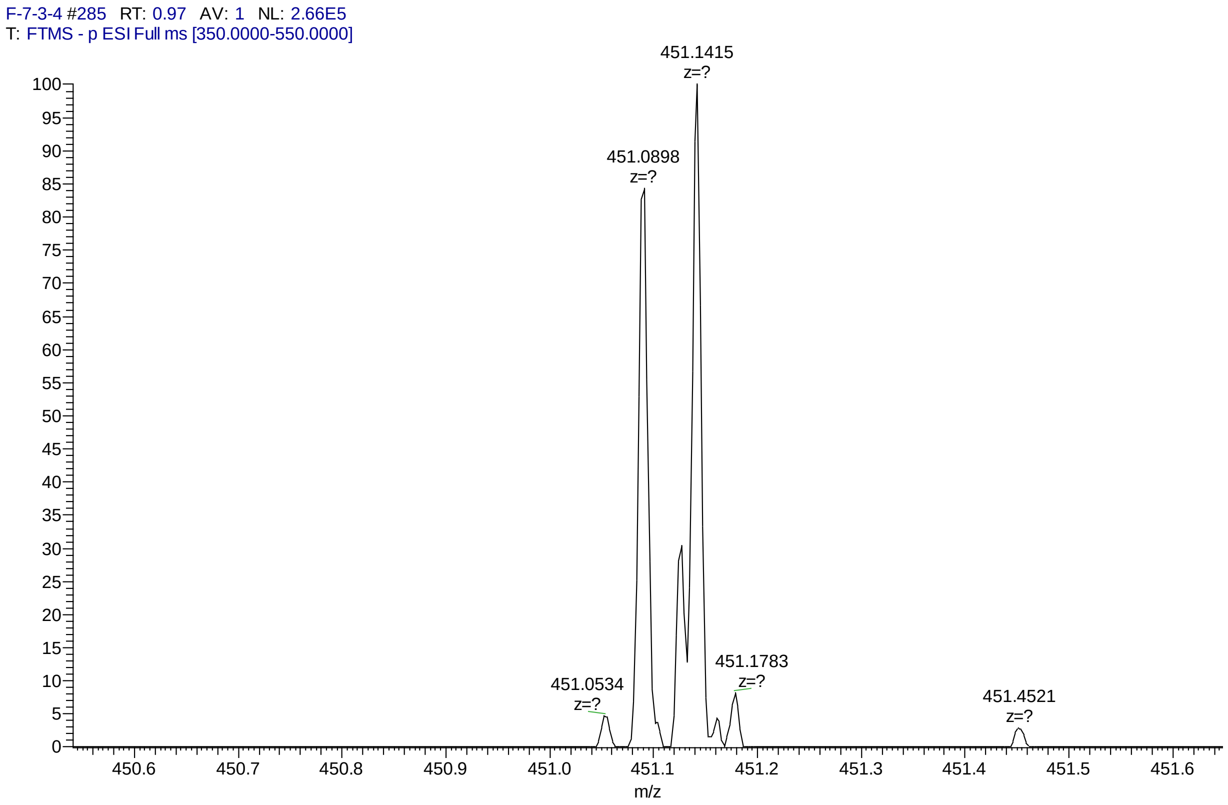

Figure S13: FT-MS spectrum for compound **II**

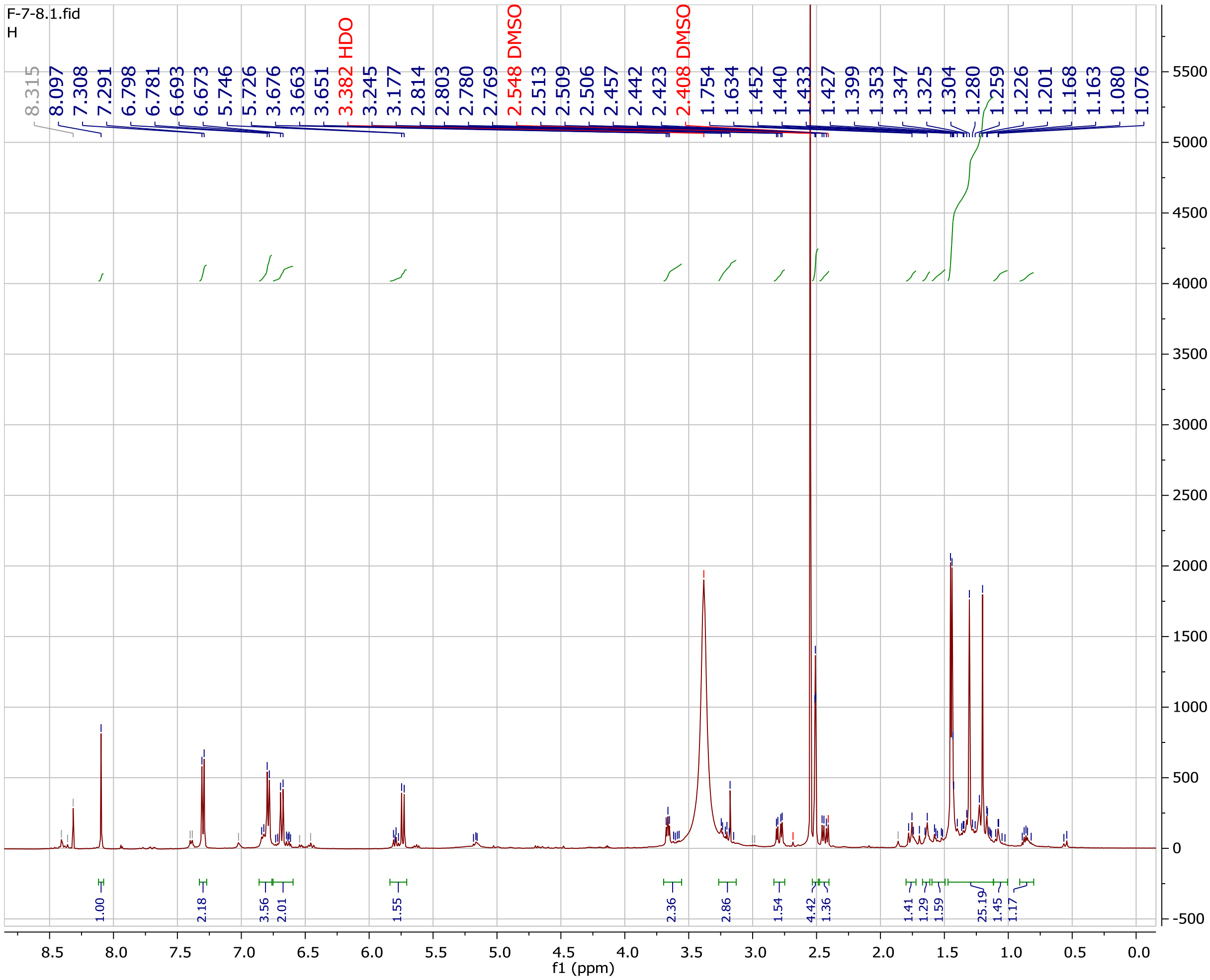

Figure S14: ^{1}H NMR spectrum for compound **III** (DMSO-*d*6, 500 MHz)

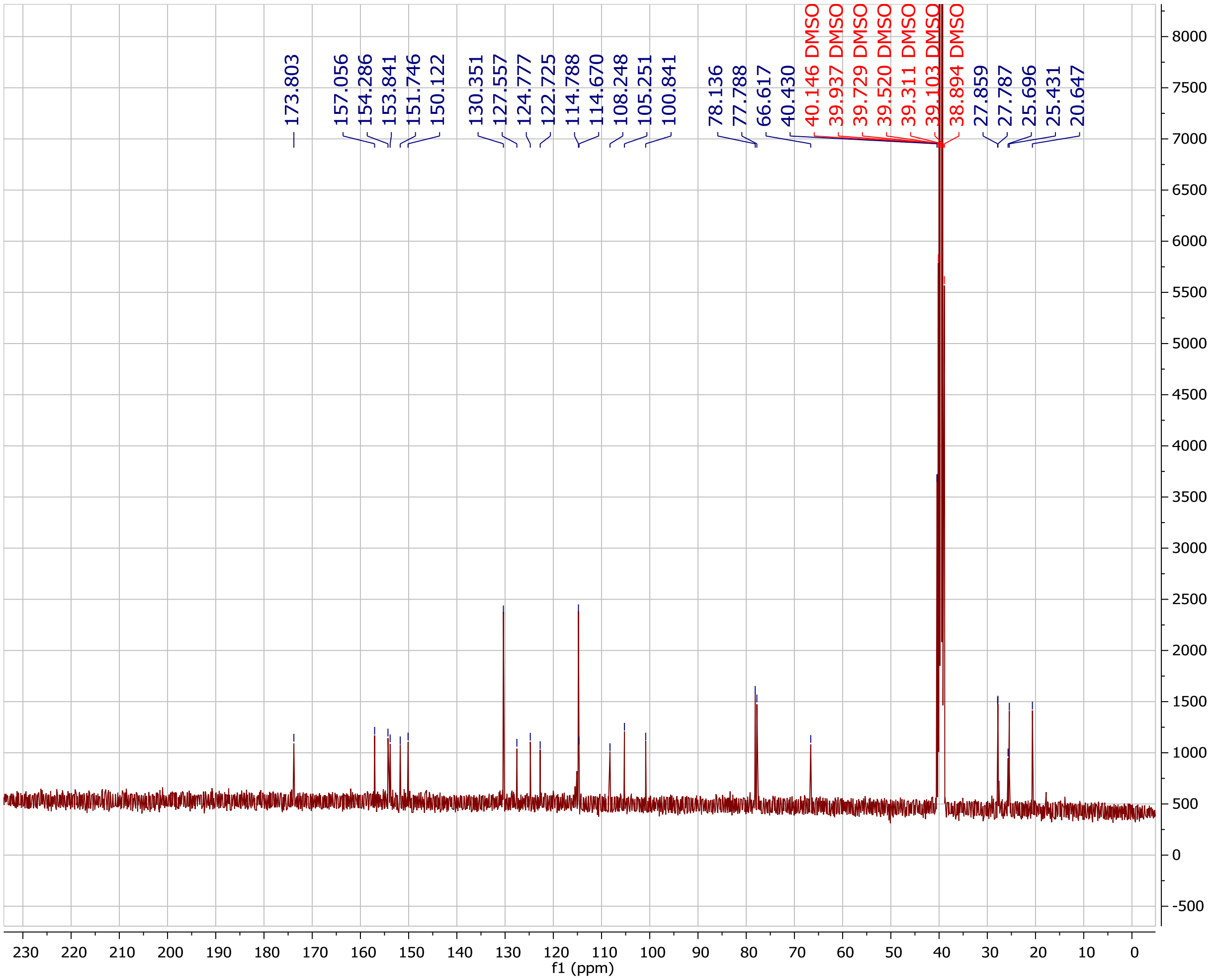

Figure S15: ^{13}C NMR spectrum for compound **III (**DMSO-*d*6, 125 MHz)

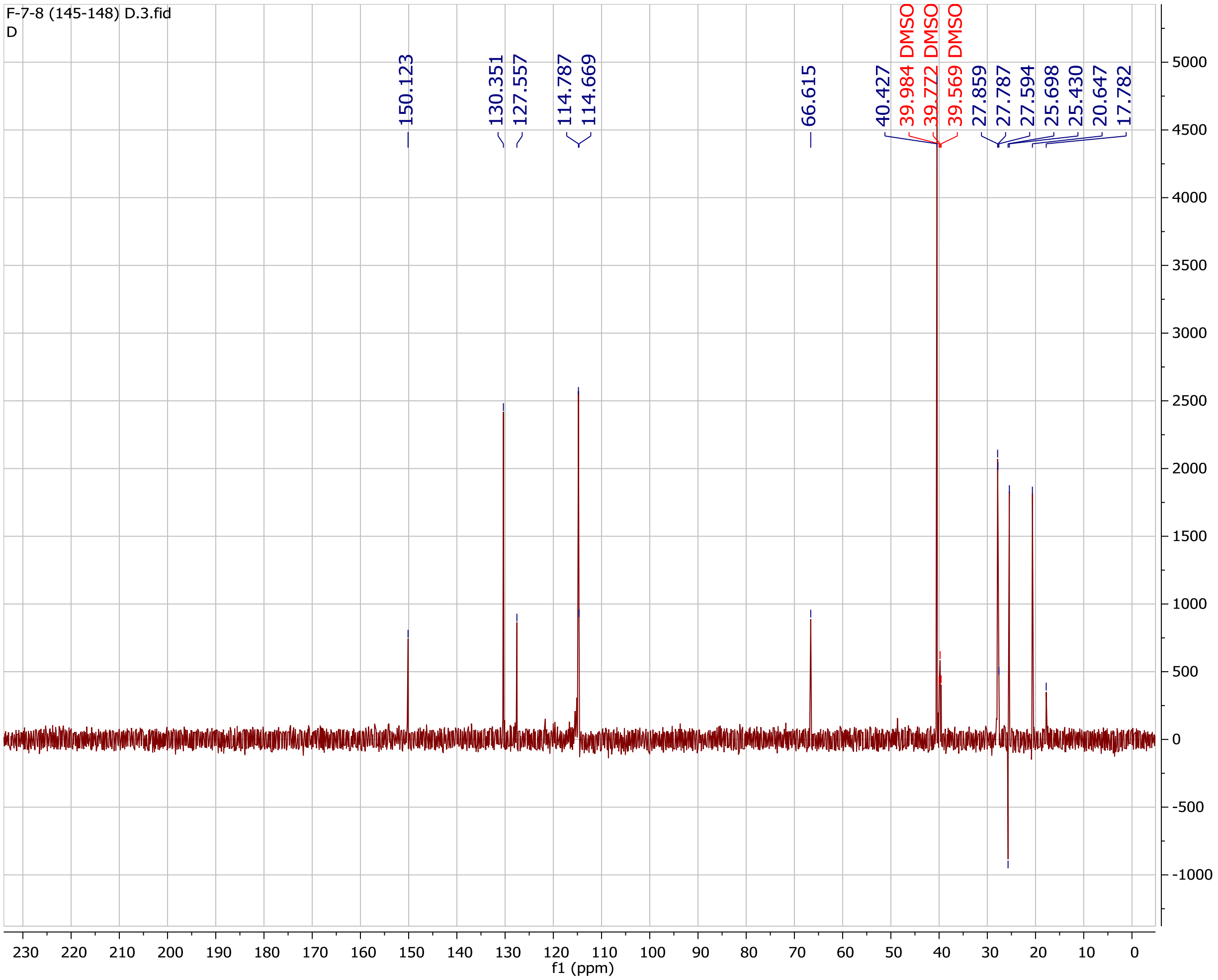

Figure S16: DEPT spectrum for compound **III (**DMSO-*d*6, 125 MHz)

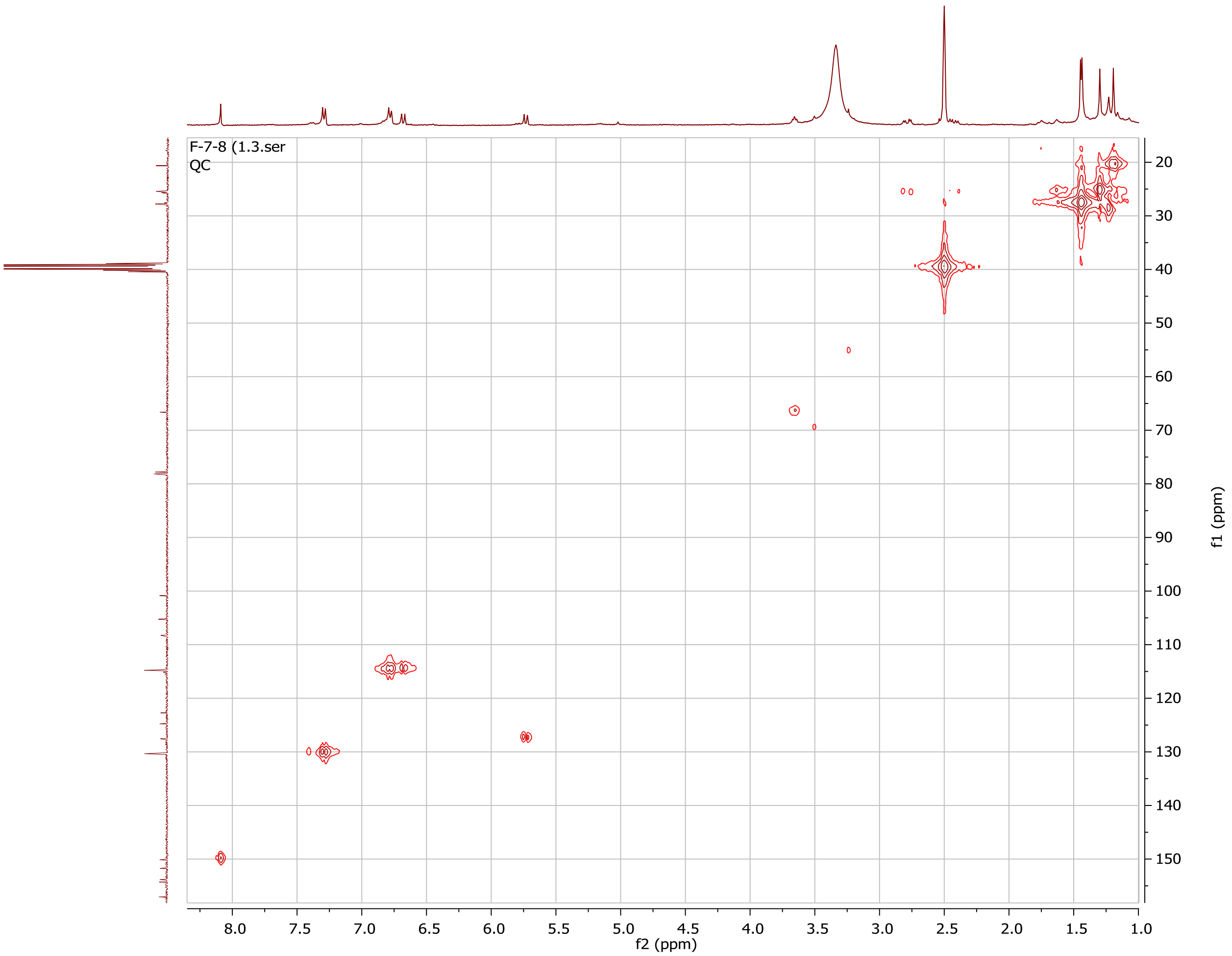

Figure S17: HSQC spectrum for compound **III** (DMSO-*d*6, 500 MHz)

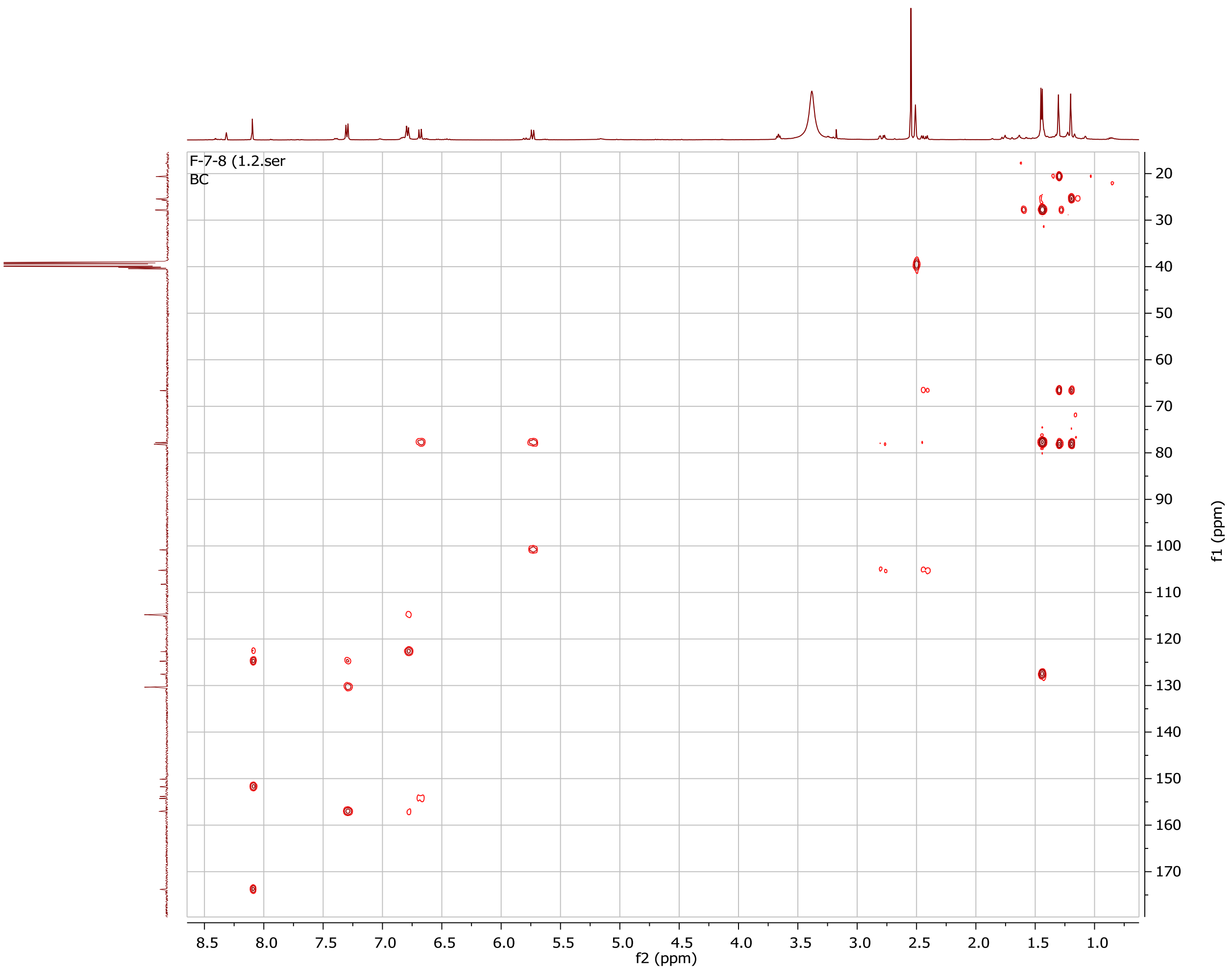

Figure S18: HMBC spectrum for compound **III** (DMSO-*d*6, 500 MHz)

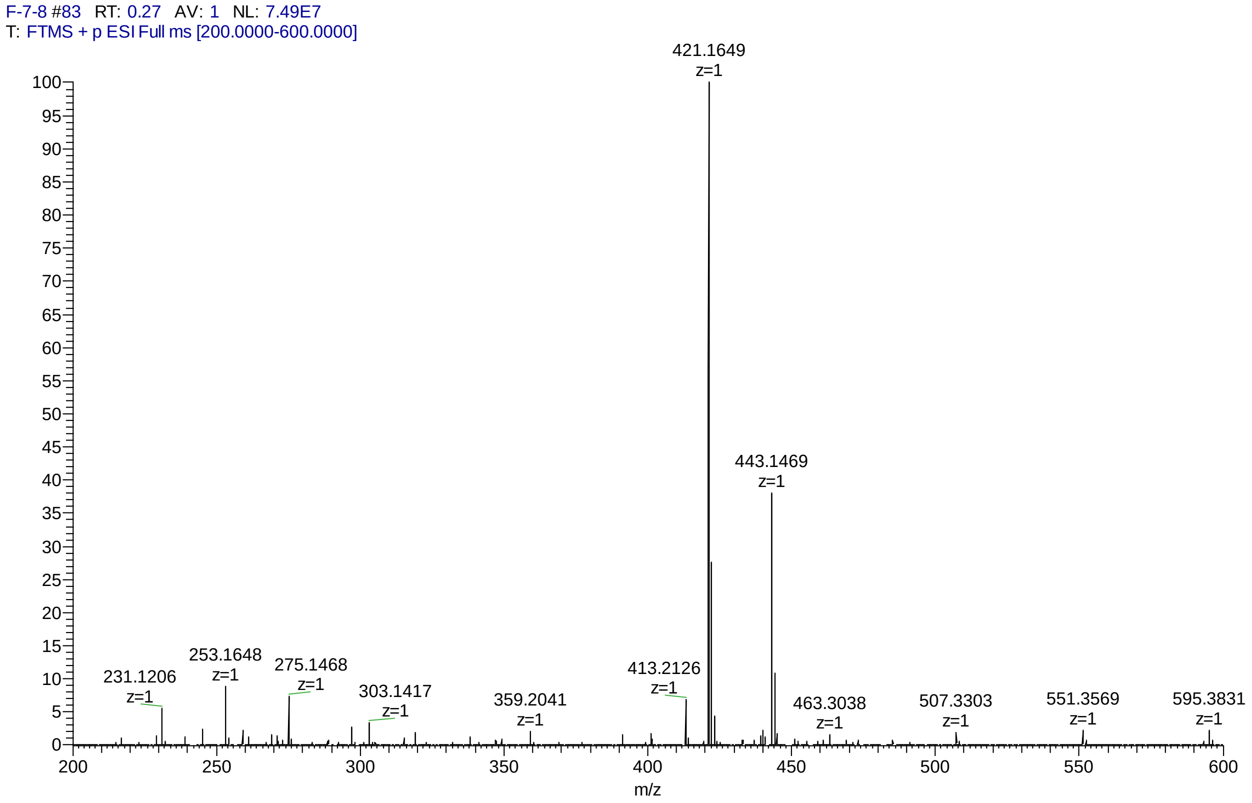

Figure S19: FT-MS spectrum for compound **III**

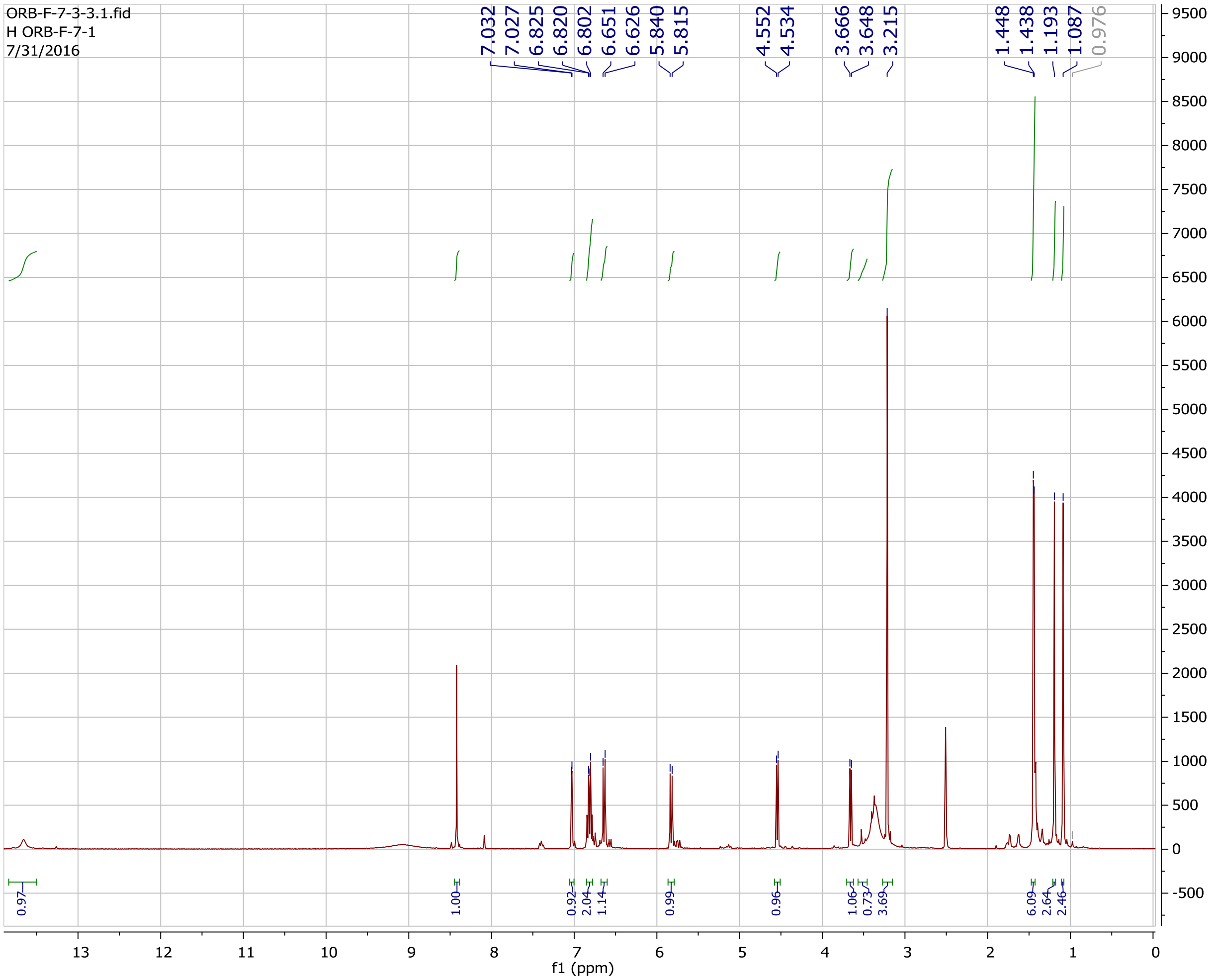

Figure S20: ^{1}H NMR spectrum for compound **IV (**DMSO-*d*6, 400 MHz)

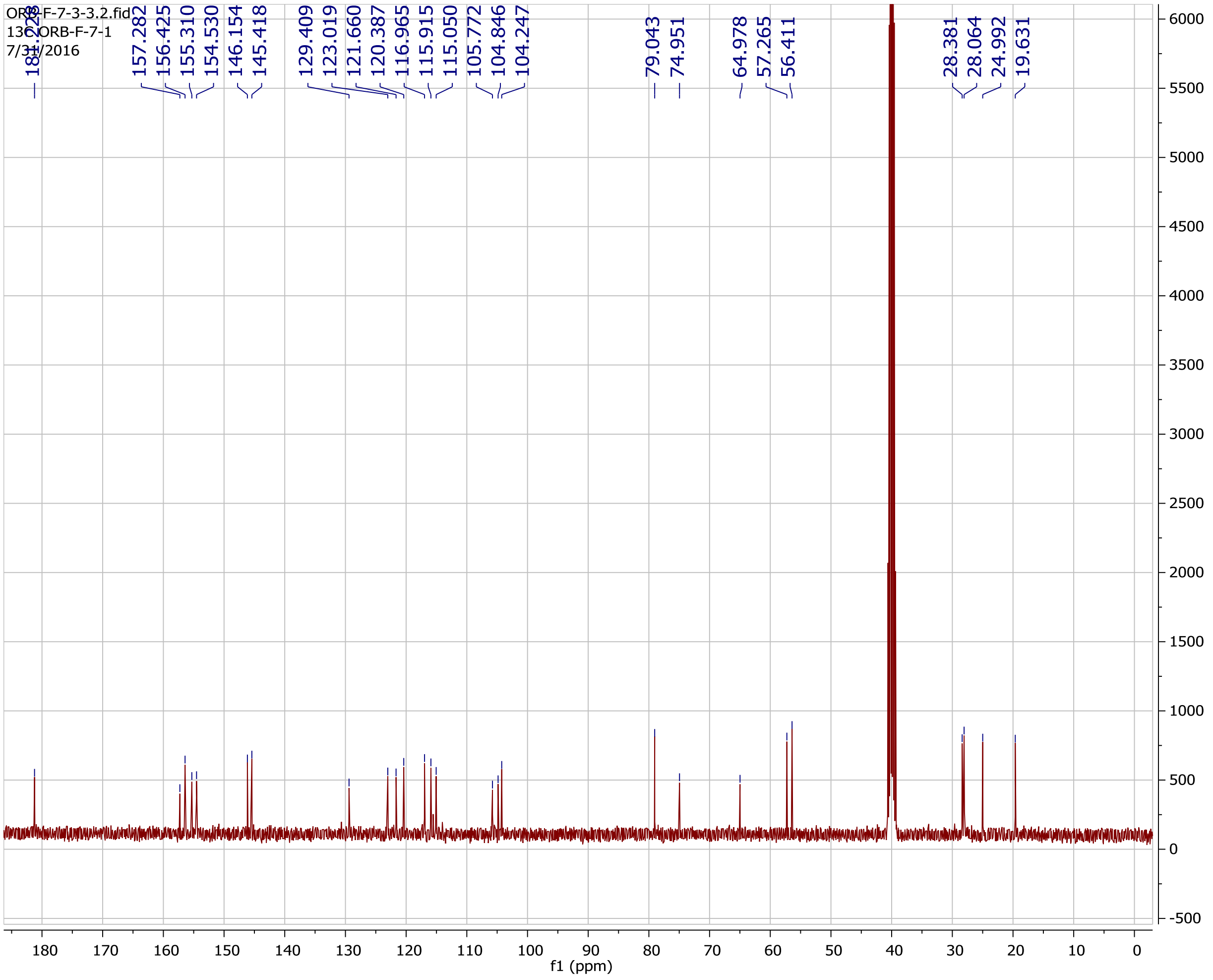

Figure S21: ^{13}C NMR spectrum for compound **IV (**DMSO-*d*6, 100 MHz)

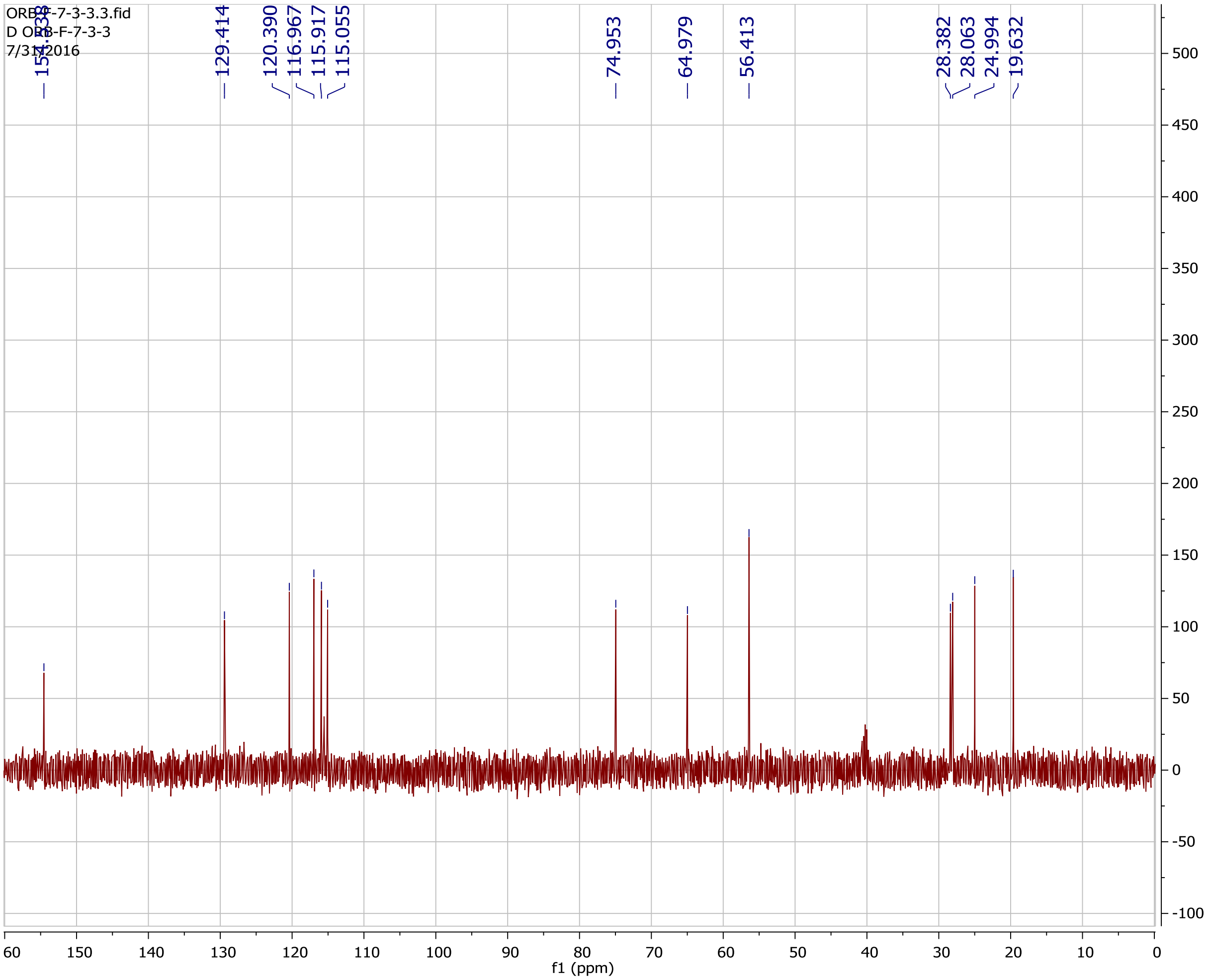

Figure S22: DEPT spectrum for compound **IV (**DMSO-*d*6, 100 MHz)

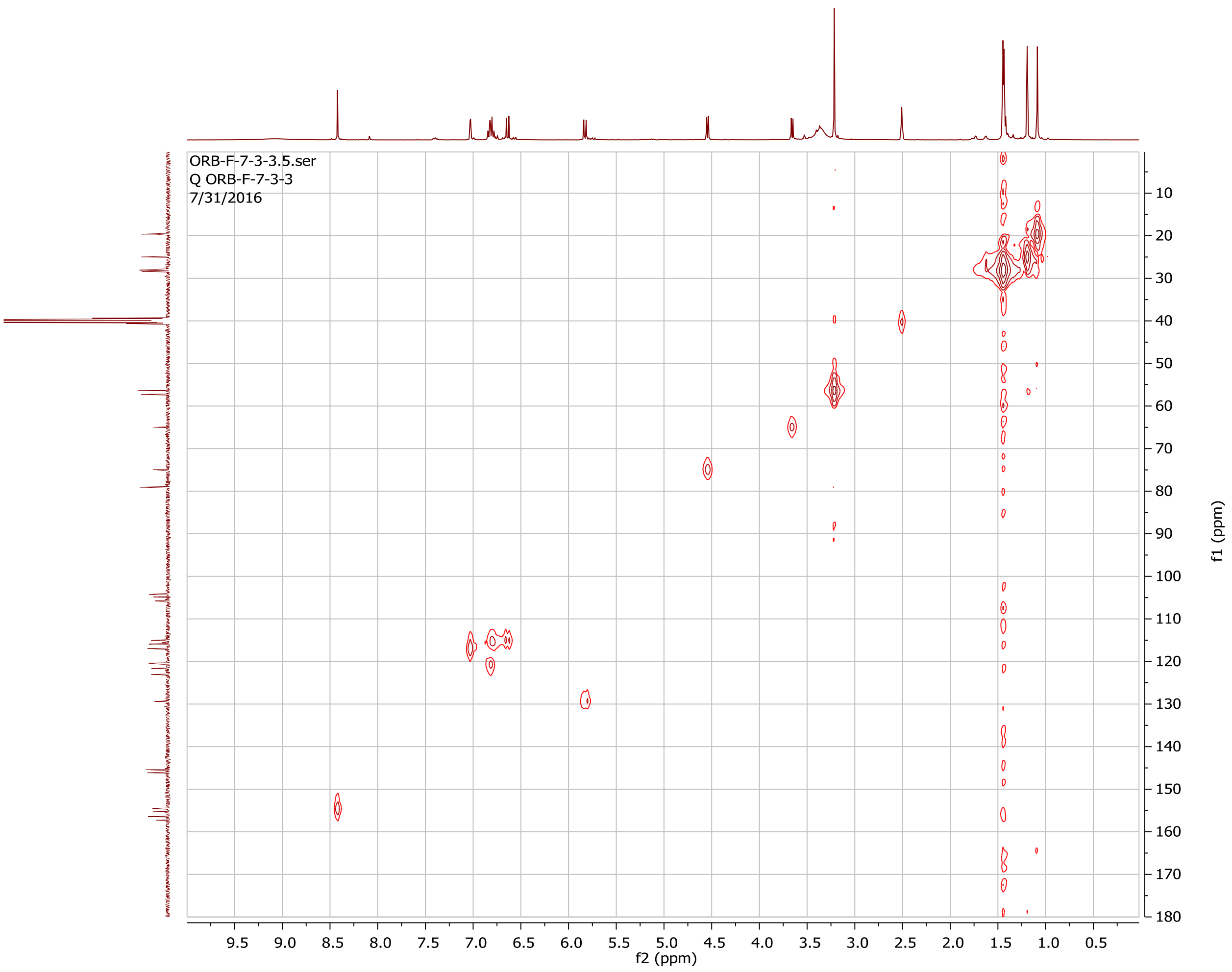

Figure S23: HSQC spectrum for compound **IV** (DMSO-*d*6, 400 MHz)

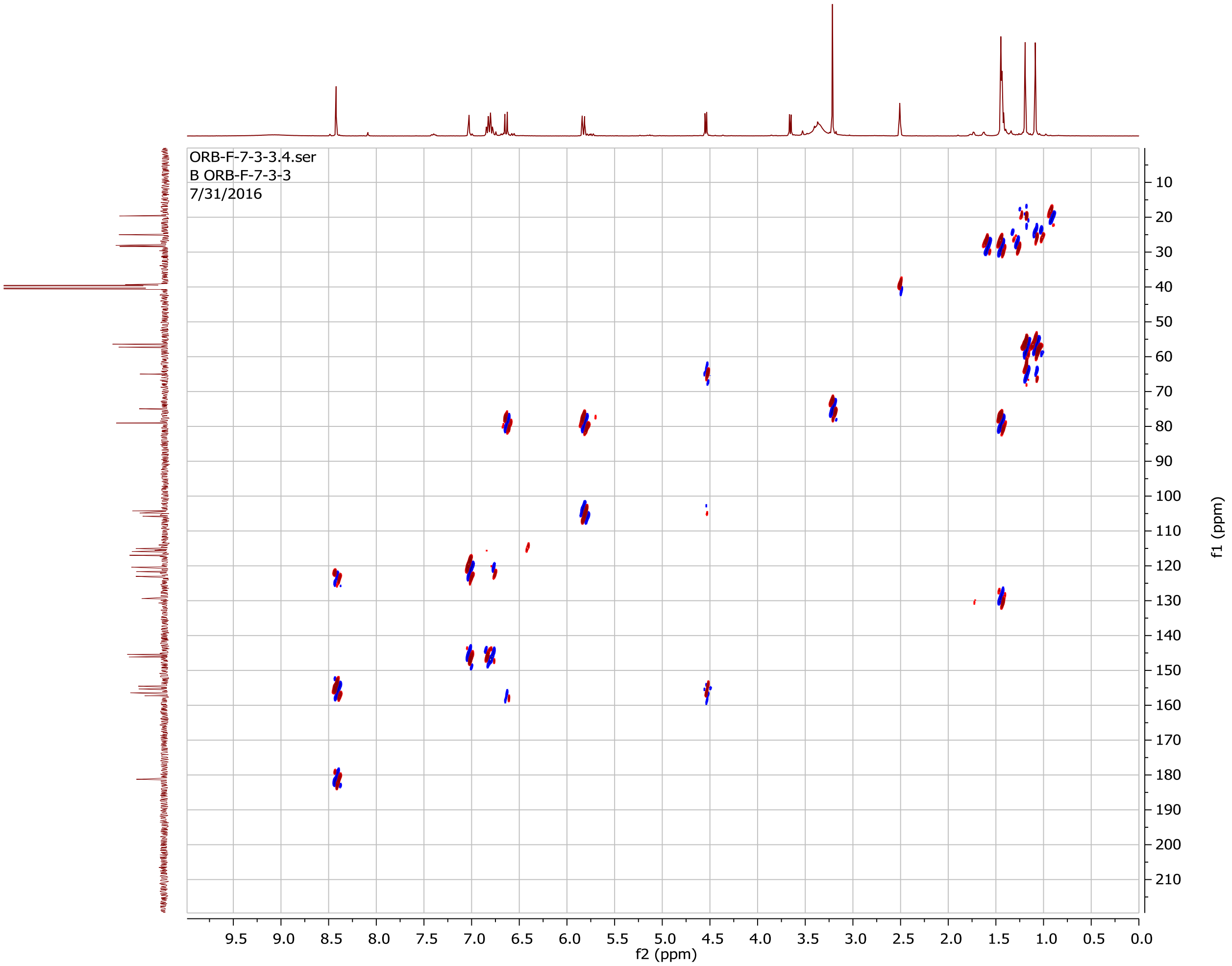

Figure S24: HMBC spectrum for compound **IV** (DMSO-*d*6, 400 MHz)

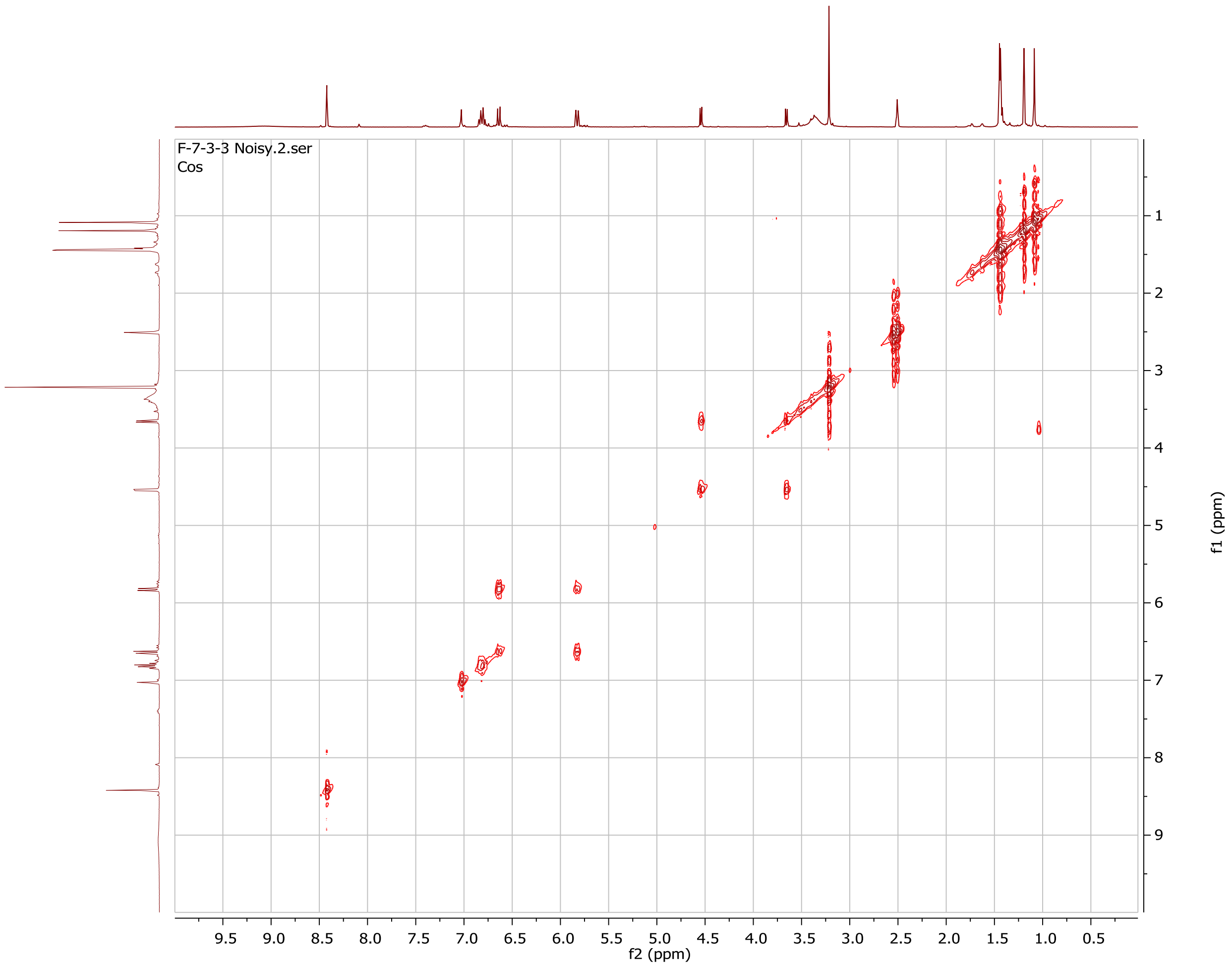

Figure S25: COSY spectrum for compound **IV** (DMSO-*d*6, 400 MHz)

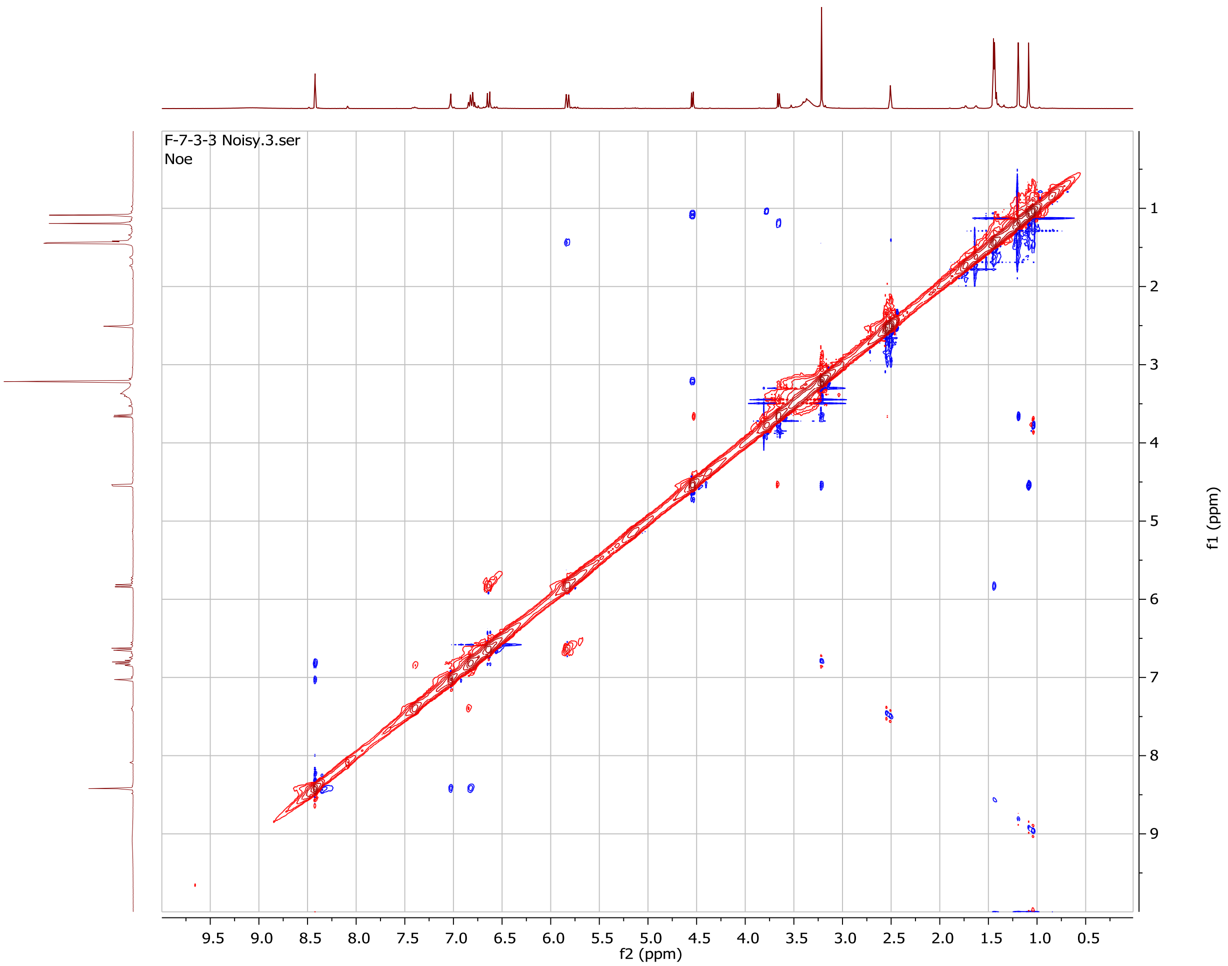

Figure S26: NOESY spectrum for compound **IV** (DMSO-*d*6, 400 MHz)

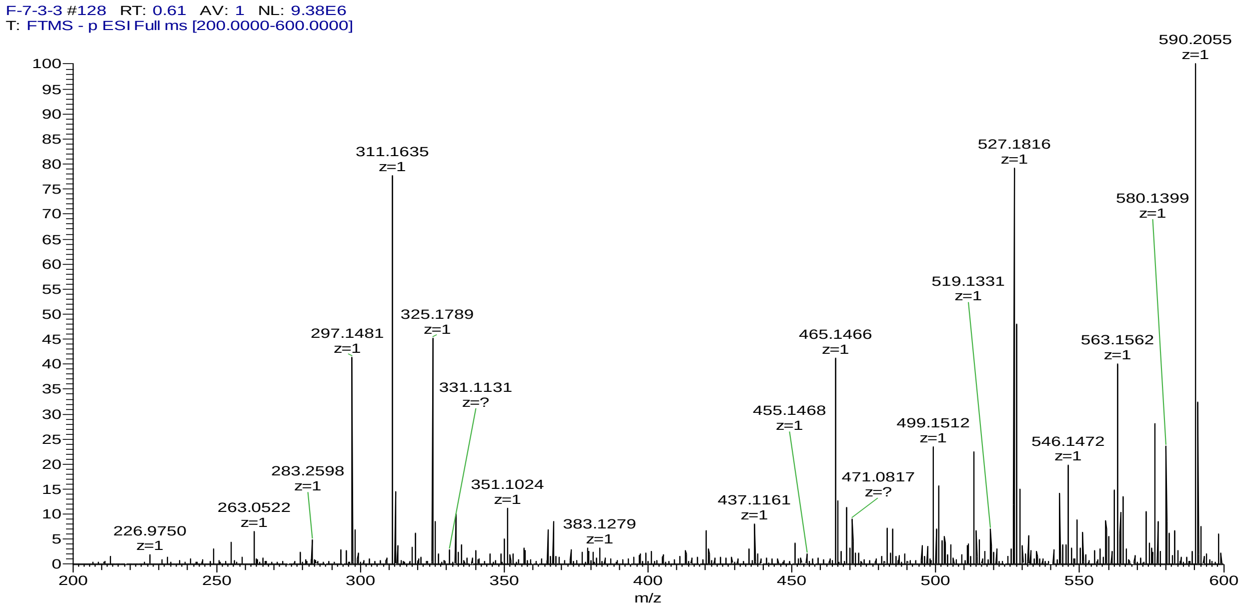

Figure S27: FT-MS spectrum for compound **IV**
